# Extracellular vesicles from human iPSCs enhance reconstitution capacity of cord blood-derived hematopoietic stem and progenitor cells

**DOI:** 10.1038/s41375-021-01325-y

**Published:** 2021-06-17

**Authors:** Elżbieta Karnas, Małgorzata Sekuła-Stryjewska, Katarzyna Kmiotek-Wasylewska, Sylwia Bobis-Wozowicz, Damian Ryszawy, Michał Sarna, Zbigniew Madeja, Ewa K. Zuba-Surma

**Affiliations:** 1 Department of Cell Biology, Faculty of Biochemistry, Biophysics and Biotechnology, Jagiellonian University, Krakow, Poland; 2 Laboratory of Stem Cell Biotechnology, Malopolska Centre of Biotechnology, Jagiellonian University, Krakow, Poland; 3 Department of Biophysics, Faculty of Biochemistry, Biophysics and Biotechnology, Jagiellonian University, Krakow, Poland

**Keywords:** Stem-cell therapies, Regenerative medicine, Haematopoietic stem cells, Cell signalling

## Abstract

Cord blood (CB) represents a source of hematopoietic stem and progenitor cells (CB-HSPCs) for bone marrow (BM) reconstitution, but clinical CB application is limited in adult patients due to the insufficient number of CB-HSCPCs and the lack of effective ex vivo approaches to increase CB-HSPC functionality. Since human-induced pluripotent stem cells (hiPSCs) have been indicated as donor cells for bioactive extracellular vesicles (EVs) modulating properties of other cells, we are the first to employ hiPSC-derived EVs (hiPSC-EVs) to enhance the hematopoietic potential of CB-derived CD45^dim^Lin^-^CD34^+^ cell fraction enriched in CB-HSPCs. We demonstrated that hiPSC-EVs improved functional properties of CB-HSPCs critical for their hematopoietic capacity including metabolic, hematopoietic and clonogenic potential as well as survival, chemotactic response to stromal cell-derived factor 1 and adhesion to the model components of hematopoietic niche in vitro. Moreover, hiPSC-EVs enhanced homing and engraftment of CB-HSPCs in vivo. This phenomenon might be related to activation of signaling pathways in CB-HSPCs following hiPSC-EV treatment, as shown on both gene expression and the protein kinases activity levels. In conclusion, hiPSC-EVs might be used as ex vivo modulators of CB-HSPCs capacity to enhance their functional properties and augment future practical applications of CB-derived cells in BM reconstitution.

## Introduction

Human umbilical cord blood (CB) is a rich source of various stem and progenitor cell types, including non-hematopoietic and hematopoietic stem and progenitor cells (HSPCs) [1]. The clinical application of CB cells for bone marrow (BM) reconstitution in patients suffering from hematological and malignant disorders is a desirable approach because of the available stem cell (SCs) content, convenient collection methods, low immunogenicity, and low risk of graft-versus-host disease [2]. This leads to an increasing number of private and public CB banks, where CB units can be donated and stored for future use [3].

However, the relatively low volume of collected CB samples implicates low number of HSPC transplantations; indeed, the wider applications of CB cells in adult patients are often limited by insufficient cell engraftment and delayed hematopoietic recovery [4]. These obstacles necessitate the optimization of current approaches and development of novel strategies for enhancing the hematopoietic activity of CB-derived cells to facilitate their homing and engraftment after transplantation [5].

Several strategies for the enhancement of CB-HSPCs functions have been proposed, including ex vivo expansion prior to cell administration, the use of various cytokine cocktails, and co-culture systems [2, 6, 7] or pre-treatment with different chemical compounds, e.g., Stem-Regenin-1 [8].

Recently, a promising approach based on paracrine cell treatment with extracellular vesicles (EVs) has been proposed [9]. In recent years, these membrane vesicles with the sizes between 50 nm and 2 µm, which can be released from different cell compartments, were isolated from various cell types and body fluids [10]. Notably, EVs were shown to harbor and transfer their bioactive content to recipient cells and to play important roles in cell-to-cell communication [11]. Consequently, there is growing interest in the utilization of EVs for clinical applications [12]. Importantly, several studies conducted by our group and others have shown that EVs released by induced pluripotent SCs (iPSCs) act as paracrine mediators influencing cell fate [13–15]. However, the possible role of EVs in the priming of CB-SCs remains unknown.

Therefore, in this study, we evaluated the impact of human iPSCs (hiPSCs)-derived EVs (hiPSC-EVs) on selected functions of CB-HSPCs in vitro and in vivo, which are important for their hematopoietic potential and BM reconstitution after myeloablation. As such, this study may provide new insight on future practical applications of CB cells in clinical practice.

## Materials and methods

For primer sequences, antibodies, and detailed experimental procedures, please refer to the Supplementary Methods.

### Primary cell cultures

Human mesenchymal stem cells (hMSCs) isolated according to a previously described explant method [16] were cultured in standard conditions (5% CO_2_, 37 °C) in DMEM/F12 medium (Sigma-Aldrich, Saint Louis, MO, USA) supplemented with 10% of fetal bovine serum (Sigma-Aldrich), penicillin, and streptomycin (100 U/ml; Thermo Fisher Scientific, Waltham, MA, USA). Human umbilical vein endothelial cells (HUVECs) were isolated using a previously described method [17] and cultured in standard conditions in EGM-2MV medium (Lonza, Basel, Switzerland).

### HSPCs isolation and expansion ex vivo

CB units were shipped from the Polish Stem Cell Bank (Warsaw, Poland), based on approvals granted to this study Partner, and processed within 24 h after collection. Written informed consent was obtained from mothers before CB donation. Fraction highly enriched in HSPCs was sorted by triple-step protocol including: (i) red blood cells lysis with BD Pharm Lyse buffer (BD Bioscience, San Jose, CA, USA), (ii) initial purification of CD34^+^ fraction using magnetic-activated cell sorter AutoMACS Pro system (Miltenyi Biotec, Bergisch Gladbach, Germany), and (iii) final re-purification by fluorescence-activated cell sorter (FACS) BD FACSAria III (BD Bioscience), following staining with antibodies against selected lineage (Lin) markers, CD45 and CD34 (BD Bioscience). HSPCs were sorted as CD45^dim^Lin^-^CD34^+^ cells. After FACS, CB-HSPCs were expanded in standard conditions in serum-free StemSpan SFEM II medium with CC110 reagent (Stem Cell Technologies, Vancouver, Canada).

### hiPSCs cultures

Native and genetically modified hiPSCs were used as donor cells for hiPSC-EV isolation. Native hiPSCs were obtained in our laboratory via reprogramming with non-integrative Sendai virus as previously described [13]. Genetically modified hiPSCs expressing green fluorescent protein cloned from copepod *Pontellina plumata* (copGFP-hiPSCs) were generated by the transduction with copGFP-expressing lentiviral vector and further purified by FACS, to produce fluorescent hiPSC-EVs.

### Preparation of hiPSC-EVs

hiPSC-EVs were isolated by sequential centrifugation including ultracentrifugation (100 000 × *g*) of the conditioned medium (CM) collected from native and modified hiPSCs, generated and cultured in serum-free, xeno-free, and feeder-free conditions, as previously described [13]. The relative amount of isolated hiPSC-EVs was assessed by protein concentration (Bradford assay) [13].

### hiPSC-EVs characterization

Particle concentration and size distribution of hiPSC-EVs were measured by NanoSight NS300 analyzer (Malvern Pananalytical, Malvern, UK) based on the nanoparticle tracking analysis (NTA). Atomic force microscopy (AFM) analysis of hiPSC-EV samples was performed by BioScope Catalyst AFM system (Brüker, Billerica, MA, USA), as previously described [13, 15]. For flow cytometry analysis, hiPSC-EVs were stained with RNASelect dye (Thermo Fisher Scientific) and antibodies against CD9, CD34, CD45, CD63, CD81, CD90, CD105, Tra-1-60, KDR, lymphocyte function-associated 1 protein (LFA-1), and stage-specific embryonic antigen-4 (SSEA-4) and further analysis was performed with an Apogee A50-Micro flow cytometer (Apogee Flow Systems, Hemel Hempstead, UK). In addition, stained hiPSC-EVs were also imaged by ImageStreamX Mk II imaging flow cytometer, using 60× objective magnification and IDEAS Software (Luminex Corp., Austin, TX, USA.). Moreover, western blotting analysis was performed to compare relative levels of expression of CD9, CD63, syntenin, calnexin and β-actin in lysates obtained from hiPSC-EVs and their parental cells.

### Incubation of CB-HSPCs with hiPSC-EVs

In all experiments, CB-HSPCs were incubated with hiPSC-EVs in the amount of 1 µg of protein per 2 × 10^5^ cells (final protein concentration of hiPSC-EVs: 2 µg/ml, corresponding to 3.03 ± 1.01 × 10^8^ particles/ml). In short-term approach experiments, CB-HSPCs were first expanded for 7 days (including day of cell isolation) and then hiPSC-EVs were added to the medium for 2, 6, 24, or 48 h. Unbound vesicles were washed out (in PBS) by centrifugation (300 *g*, 5 min, RT). In the long-term approach, cells were expanded in the medium containing hiPSC-EVs for 14 days. Every second day, a fresh portion of hiPSC-EVs was added. In both approaches cells cultured in the medium without hiPSC-EVs served as a control. To assess proliferation rate, CB-HSPCs were counted immediately after FACS isolation (0 day) and on further 4, 6, 11, and 14 days of their expansion (day of cell isolation was not counted).

### Fluorescence microscopy

To visualize the uptake of hiPSC-EVs into CB-HSPCs, cells were incubated with copGFP+ hiPSC-EVs for 2 h and subsequently subjected to the fluorescence microscopy analysis using Leica DMI6000B microscope (DMI7000 version; Leica Microsystems, Wetzlar, Germany).

### Metabolic activity assessment

Expanded CB-HSPCs were incubated with hiPSC-EVs for 2, 24, and 48 h. The concentration of ATP produced by the cells was further measured by the ATPLite Luminescence Assay System (Perkin Elmer), according to the manufacturer’s protocol.

### Analysis of cell phenotype

At the indicated time points of the ex vivo expansion (4, 6, 11, and 14 days), CB-HSPCs were harvested and stained with antibodies against CD34 or selected Lin markers. Next, the analysis of antigen expression was performed with LSRFortessa flow cytometer (BD Bioscience).

### Colony-forming cell (CFC) assay

CB-HSPCs treated with hiPSC-EVs were seeded in Human Methylcellulose Enriched Medium (R&D Systems, Minneapolis, MN, USA) and cultured for 14 days. Hematopoietic colonies were counted under Olympus IX81 microscope (Olympus Corp., Tokyo, Japan).

### Apoptosis assay

Expanded CB-HSPCs were incubated with hiPSC-EVs for 2 h and then staurosporine (100 nM; Sigma-Aldrich) was added to the culture medium to induce cell apoptosis. Cells were stained with Annexin V (Anx V) Apoptosis Detection Kit (BD Bioscience) after 24 h, according to the manufacturer’s protocol and subjected into the flow cytometry analysis.

### Chemotaxis assay

Expanded CB-HSPCs were incubated with hiPSC-EVs for 2, 6, and 24 h. Next, cells were seeded onto transwell inserts with medium containing stromal cell-derived factor 1 (SDF-1) (100 ng/ml; Peprotech, Rocky Hill, NJ, USA) in lower chamber. Transmigrated cells were counted after 3 h by flow cytometry. Before seeding, a part of the cells was examined for C-X-C motif chemokine receptor 4 (CXCR4) expression by flow cytometry.

### Calcium flux

Expanded CB-HSPCs were incubated with hiPSC-EVs for 2, 6, and 24 h and then loaded with Fluo-4 calcium indicator (Thermo Fisher Scientific). SDF-1-induced signal from Fluo-4, indicating calcium flux, was immediately measured by flow cytometry.

### Adhesion assay

Expanded CB-HSPCs were incubated with hiPSC-EVs for 2 h and stained with calcein AM (Vybrant Cell Adhesion Assay kit; Thermo Fisher Scientific), according to the manufacturer’s protocol. Next, cells were seeded onto 96-well culture plates either coated with fibronectin (50 µg/ml, Sigma-Aldrich) or covered with the monolayer of hMSCs or HUVECs. Unattached cells were washed out after 2.5 h and the fluorescent signal from remaining CB-HSPCs was measured by Infinite M200 Pro analyzer (Tecan, Männedorf, Switzerland). In addition, images of the attached cells were captured using fluorescent microscopy.

### Expression of adhesion markers

Expanded CB-HSPCs were incubated with hiPSC-EVs for 2, 6, and 24 h, stained with the fluorescently labeled antibodies against CD49d, CD49e, and LFA-1. Next, the level of antigen expression was measured by flow cytometry.

### Gene expression

In the short-term approach, expanded CB-HSPCs were incubated with hiPSC-EVs for 2, 6, or 24 h. The detection of mRNA expression for *SCL*, *HOXB4*, and *BCL-2* genes was assessed using quantitative real-time PCR (primer sequences are listed in Supplementary Table 1).

In the long-term approach, freshly isolated CB-HSPCs were cultured for 4 or 8 days in the presence of hiPSC-EVs and analyzed with Hematopoiesis RT^2^ Profiler PCR Arrays (Qiagen, Hilden, Germany), according to the manufacturer’s protocol (analyzed genes are listed in Supplementary Table 2). Relative differences in gene expression were visualized by the Heatmapper tool (http://www.heatmapper.ca/). The NetworkAnalyst tool (http://www.networkanalyst.ca) was used for the graphical representation of selected gene interactions, with the implementation of the STRING Interactome database and confidence cutoff score of 900.

### Semiquantitative assessment of kinase activity

Expanded CB-HSPCs were incubated with hiPSC-EVs for 2 h and then subjected into the relative analysis of 43 protein phosphorylations (listed in Supplementary Table 3), using Proteome Profiler Human Phospho-Kinase Array Kit (R&D Systems), according to the manufacturer’s protocol. The obtained data were analyzed with PANTHER (Protein Analysis Through Evolutionary Relationships) Pathway Classification System and STRING tool (Search Tool for the Retrieval of Interacting Genes/Proteins).

### In vivo BM reconstitution assay

All in vivo procedures were approved by the 2nd Local Institutional Animal Care and Use Committee in Krakow (Resolution No 137/2016) and conform to the relevant regulatory standards. Expanded CB-HSPCs were incubated with hiPSC-EVs for 2 h and transplanted by retro-orbital plexus injection into male γ-irradiated 8–10-week old diabetic–severe combined immunodeficiency disease (NOD/SCID) mice (2 × 10^5^ of cells/mouse), randomly assigned to the groups. Investigators were not blinded. The number of animals was chosen based on the previous studies [15]. Animals were sacrificed 48 h (analysis of homing) or 8 weeks (analysis of engraftment) post transplantation. BM, peripheral blood (PB), and spleen were harvested and stained with anti-human CD45 antibody, to evaluate the presence of human hematopoietic (hCD45^+^) cells by flow cytometry. In addition, BM cells were subjected into CFC assay to assess clonogenic potential of transplanted cells.

### Statistical analysis

Unless otherwise stated, all experiments were repeated at least three times. If more, the number of repeats was increased due to the possible observed variability between the CB donors. Data are presented as mean ± standard deviation (SD). Statistical significance was calculated using two-tailed Student’s *t*-test, with the value of *p* < 0.05 considered as significant, as indicated in the figure legends. Variance was comparable between groups in the same test. No samples or animals were excluded from the analyses.

## Results

### hiPSC-EVs consist of small vesicles with bioactive content

**Fig. 1:**
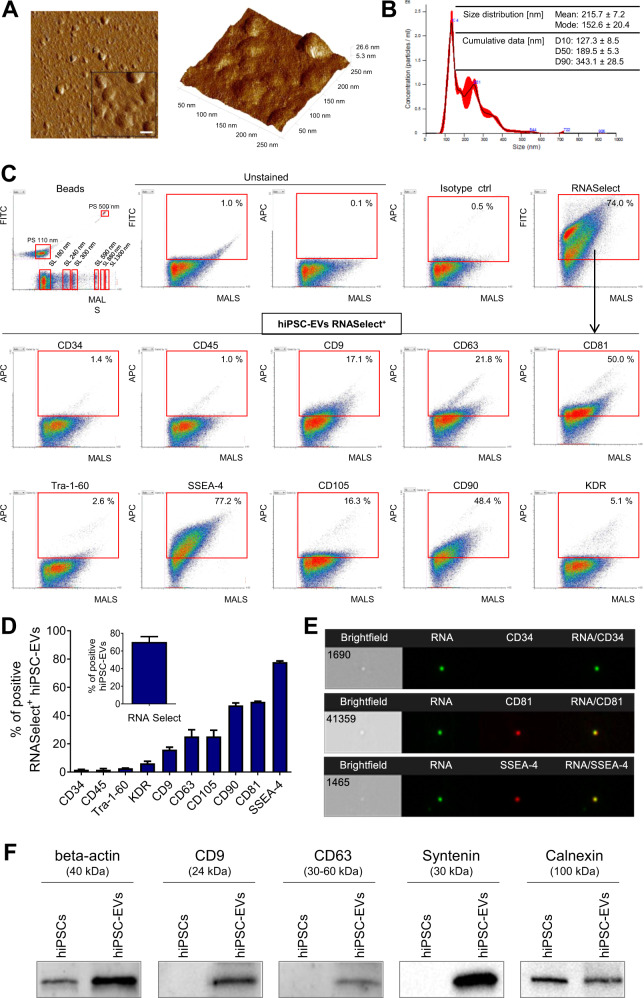
Characterization of hiPSC-EVs. **A** Representative atomic force microscopy (AFM) images of hiPS-derived EVs. Left: 2D image projection. The scale bar in the enlarged area indicates 50 nm. Right: 3D topography view of analyzed EVs with scan area 250 × 250 nm. **B** Nanoparticle tracking analysis of hiPSC-EVs samples. An exemplary particle size distribution plot obtained from sample measurement. The table contains data obtained from experimental repetitions and is presented as the mean ± SD (*N* = 3). The cumulative parameters D10, D50, and D90 indicate that 10%, 50% or 90% of the particles, respectively, have a diameter less than or equal to the given value. **C** Flow cytometry phenotyping. Representative dot plots of hiPSC-EVs samples stained with RNASelect dye and fluorescent antibodies were acquired by Apogee A50-Micro flow cytometer. The mixture of size-defined green polystyrene (PS) and silica (SL) Apogee calibration beads is shown as a size distribution reference. In gating strategy, only objects positive for RNASelect were included in the analysis of surface antigen expression. The percentage of objects positive for the analyzed markers is shown in red gates. MALS-medium angle light scatter parameter, corresponding to the relative size of analyzed particles. **D** Quantitative data for hiPSC-EVs phenotyping and presented as the mean ± SD from experimental repetitions (*N* = 3). **E** ImageStream Mk II analysis. Exemplary images of single hiPSC-EVs stained with RNASelect and selected antibodies, captured in brightfield and fluorescence channels. 60× magnification objective was used for the sample acquisition. **F** Western blotting analysis. Representative membrane images showing the expression of exosomal markers (CD9, CD63, syntenin), β-actin, and endoplasmic reticulum protein (calnexin) in hiPSC-EVs and their parental cells.

First, we performed extensive analyses of hiPSC-EV samples obtained by the sequential centrifugation of CM from hiPSCs. We confirmed their vesicular structure (Fig. 1A) a mean size as 215.7 ± 7.2 nm (Fig. 1B), based on AFM and NTA measurements, respectively. Moreover, high-resolution flow cytometry (Fig. 1C, D) revealed that hiPSC-EVs possess a high RNA content (68.15 ± 7.28%), as indicated by RNASelect dye staining. We assumed that the RNASelect^+^ population represents intact vesicular structures. These RNASelect^+^ hiPSC-EVs possessed the exosomal tetraspanins CD9 (15.85 ± 1.77%), CD63 (25.20 ± 4.81%), and CD81 (49.70 ± 0.42%), as well as differential presence of pluripotency-related markers: SSEA-4 (77.80 ± 0.85%) and Tra-1-60 (2.70 ± 0.14%). Finally, other antigens abundant on hiPSCs [13] were also found on their EVs, including KDR (6.15 ± 1.48%), CD90 (47.15 ± 1.77%), and CD105 (25.20 ± 4.38%), with lack of hematopoietic markers: CD34 (1.60 ± 0.28%) and CD45 (1.60 ± 0.85%). The percentage of hiPSC-EVs positive for analyzed surface antigens was generally lower within RNASelect^-^ population, when compared to RNASelect^+^ fraction (Supplementary Fig. 1). In addition, imaging flow cytometry confirmed hiPSC-EVs phenotype (Fig. 1E). The expression of tetraspanins, as well as syntenin on hiPSC-EVs was also demonstrated by western blotting (Fig. 1F). Interestingly, endoplasmic reticulum protein calnexin, considered as a negative marker for exosomes [18], was also detected, but its expression was lower than in hiPSCs lysate (Fig. 1F). This indicates the presence of not only exosomes but also larger microvesicles in the hiPSC-EV samples [18, 19].

Collectively, we confirmed that hiPS-EVs contain a heterogeneous population of vesicles that fulfill International Society for Extracellular Vesicles criteria of EV definition [18, 20].

### hiPSC-EVs can enter CB-HSPCs

**Fig. 2:**
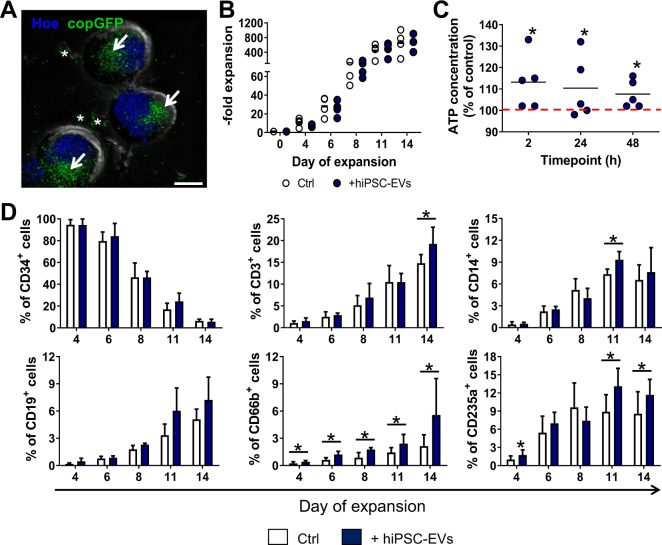
hiPSC-EVs target CB-HSPCs during their ex vivo expansion. CD34^+^ cell population highly enriched in HSPCs was purified from CB units and cultured in a dedicated expansion medium containing hiPSC-EVs. **A** Visualization of hiPSC-EVs internalization into CB-derived CD34^+^ cells. Cells were incubated with copGFP+ hiPSC-EVs for 2 h. After the removal of unbound hiPSC-EVs, cell nuclei were stained with Hoechst 33342 dye (Hoe). Cell images were captured by Leica DMI6000B microscope with 100× NA-1.47 oil immersion objective. A representative merged image for differential interference contrast module and fluorescence channels for copGFP and Hoe is presented. White arrows indicate copGFP+ hiPSC-EVs accumulated inside the CB-HSPCs. White asterisks indicate aggregated copGFP+ hiPSC-EVs attached to the cells. Scale bar indicates 5 µm. **B** Kinetics of CB-HSPCs ex vivo expansion. Data are expressed as fold expansion compared to the number of isolated CB-HSPCs. Each dot represents data obtained from individual experimental repetition (*N* = 4) for cells expanded in the control medium (Ctrl) or hiPSC-EVs (+hiPSC-EVs) medium. **C** The effect of hiPSC-EVs on the metabolic activity of CB-derived HSPCs. Expanding CB-HSPCs were treated with hiPSC-EVs for 2, 24, or 48 h. Subsequently, a luminescence assay was performed to measure the concentration of ATP produced by the cells. Data on the graph present the ATP level in hiPSC-EVs-treated cells expressed as the percentage of the control (cells untreated with hiPSC-EVs) in individual experimental repetitions (*N* = 5). Black lines represent the mean value, whereas the red line indicates the level of the control (100%). *p < 0.05 for the control vs. hiPSC-EVs-treated cells, two-tailed Student’s *t*-test. **D** Kinetics of phenotypic changes in CB-HSPCs during ex vivo expansion. Cells were expanded for 14 days in control medium (Ctrl) or medium containing hiPSC-EVs (+hiPSC-EVs). On the indicated day of the expansion, cells were harvested and stained with fluorescent-conjugated antibodies against CD34 and hematopoietic lineage markers. The analysis of antigen expression was performed with the BD LSRFortessa flow cytometer. Data are presented as mean ± SD (*N* = 3). *p < 0.05 for control vs. hiPSC-EVs-treated cells, two-tailed Student’s *t*-test.

As we have previously demonstrated, hiPSC-EVs may interact with primary cardiac cells, delivering their bioactive content and influencing functions of recipient cells [13]. Thus, we tested whether hiPSC-EVs can interact with CB-derived fraction highly enriched in HSPCs [21, 22], which was purified by double sorting protocol, including MACS followed by FACS (Supplementary Fig. 2A), reaching the final purity of CD45^dim^Lin^-^CD34^+^ population >98% (Supplementary Fig. 2B). Next, we incubated cells ex vivo with copGFP^+^ hiPSC-EVs and using fluorescent microscopy we visualized copGFP+ hiPSC-EVs that entered CB-HSPCs and localized perinuclearly in the cytoplasm (Fig. 2A and Supplementary Movie 1), with few EV aggregates surrounding cells. This result demonstrates that hiPSC-EVs may enter and be internalized into CB-HSPCs cells.

### hiPSC-EVs do not increase the proliferation of CB-HSPCs, but enhance their metabolic activity

Despite effective proliferation of CB-HSPCs in a dedicated serum-free medium (Fig. 2B), their ex vivo expansion was time-restricted, with changes in cell morphology (Supplementary Fig. 3A) and progressive decrease in population doubling time (Supplementary Fig. 3B). In addition, we did not observe enhancing effect of hiPSC-EVs on CB-HSPCs proliferation (Fig. 2B). However, short incubation with hiPSC-EVs elevated the ATP production-related metabolic activity of these cells, with the highest effect for cells treated with hiPSC-EVs for 2 h (Fig. 2C). Thus, these results indicate that hiPSC-EVs can transiently activate the metabolic activity of CB-HSPCs cells, with no significant effect on their ex vivo expansion rate.

### hiPSC-EVs enhance spontaneous differentiation of CB-HSPCs

Despite use of expansion medium containing early-acting cytokines, known to activate stem and immature progenitor cell cycling rather than their differentiation [23], we observed changes in CB-HSPCs morphology during ex vivo expansion, which could indicate hematopoietic differentiation (Supplementary Fig. 3A). Indeed, evaluation of phenotype kinetics demonstrated gradual decrease of CD34 antigen expression and increase in the percentage of cells positive for particular lineage markers related to hematopoietic maturation, including CD3, CD14, CD19, CD66b, and CD235a, that corresponds to T and B cells, monocyte-macrophage, and erythroid lineages, respectively (Fig. 2D and Supplementary Table 4). Moreover, the addition of hiPSC-EVs to the expanding medium did not influence CD34 expression but had an effect on lineage differentiation of CB-HSPCs, with special regard to increase in CD66b and CD235a expression (Fig. 2D and Supplementary Table 4). Thus, we concluded that hiPSC-EVs might stimulate spontaneous differentiation of CB-HSPCs during ex vivo expansion.

### Clonogenic potential of CB-HSPCs increases upon hiPSC-EVs treatment

**Fig. 3:**
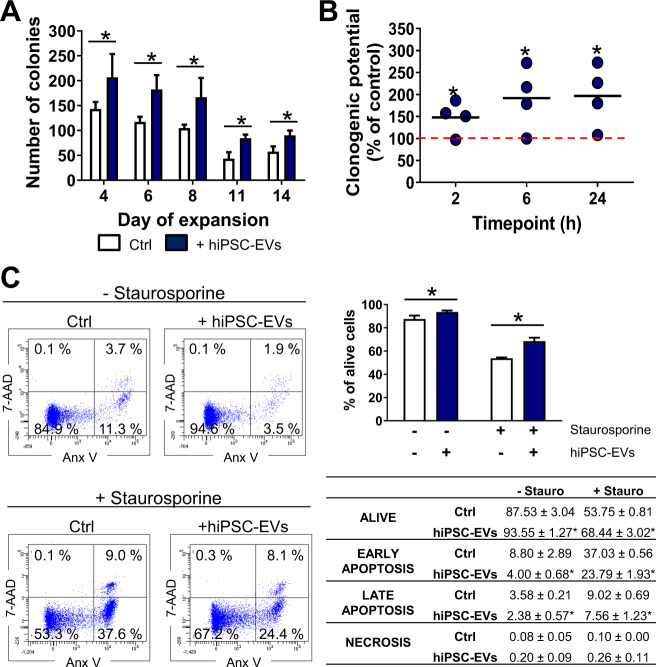
The effect of hiPSC-EVs on hematopoietic functions of CB-HSPCs in vitro. **A** Clonogenic potential of CB-HSPCs expanded with hiPSC-EVs. Cells were cultured for 14 days in the control medium (Ctrl) or hiPSC-EVs medium (+hiPSC-EVs). On selected days of expansion 5 × 10^2^ cells were seeded in a dedicated methylcellulose-based medium for colony-forming cell (CFC) assays. Hematopoietic colonies were counted after 14 days. The total number of colonies generated by the cells seeded for CFC assays on the indicated day of expansion is presented as mean ± SD (*N* = 3). *p < 0.05 for control vs. hiPSC-EVs-treated cells, two-tailed Student’s *t*-test. **B** The effect of short-term treatment with hiPSC-EVs on the clonogenic potential of expanded cells. Prior experiment, cells were expanded for 7 days in the control medium without hiPSC-EVs and were subsequently treated with hiPSC-EVs for 2, 6, or 24 h. Data on the graph present the clonogenic potential of hiPSC-EVs-treated cells expressed as the percentage of the control (cells untreated with hiPSC-EVs) in individual experimental repetitions (*N* = 4). Black lines represent the mean value, whereas the red line indicates the level of the control (100%). *p < 0.05 for control vs. hiPSC-EVs-treated cells, two-tailed Student’s *t*-test. **C** The cytoprotective effect of hiPSC-EVs on CB-HSPCs. Prior experiment, cells were expanded for 7 days in the control medium without hiPSC-EVs and were then treated with hiPSC-EVs for 2 h. Subsequently, staurosporine (100 nM) was added to the culture medium for 24 h. Cell viability was assessed by Annexin V Apoptosis Detection Kit and BD LSRFortessa flow cytometer. In gating strategy, live (Anx V- 7-AAD-), early-apoptotic (Anx V+ 7-AAD-), late apoptotic (Anx V+ 7-AAD+), and necrotic cells (Anx V- 7-AAD+) cells were analyzed. Left panel: representative dot plots with the percentage of cells present in individual gates. Right panel: summarized data of cell viability presented as mean ± SD (*N* = 4). Data on the graph demonstrate the percentage of live cells. The table summarizes the percentage of cells in particular cell death phases. *p < 0.05 for control vs. hiPSC-EVs-treated cells, two-tailed Student’s *t*-test.

As the clonogenic potential of HSPCs is an important factor influencing the effectiveness of hematopoietic system reconstitution after cell transplantation [24], we performed CFC assay to test the influence of hiPSC-EVs on the ability of the CB-HSPCs population to form different types of hematopoietic colonies (Supplementary Fig. 4A). Consistent with the depletion of CD34 antigen expression, clonogenic potential of expanding cells gradually decreased. However, addition of hiPSC-EVs to expansion medium resulted in significantly more colonies that were formed (Fig. 3A and Supplementary Fig. 4B). Similarly, clonogenic potential was higher for expanded CB-HSPCs treated with hiPSC-EVs for 2, 6, or 24 h (Fig. 3B and Supplementary Fig. 4C). Collectively, these data demonstrate that both long-term and short-term treatment with hiPSC-EVs enhance the clonogenic potential of CB-HSPCs.

### hiPSC-EVs exhibit a cytoprotective effect on CB-HSPCs

As our previous studies have shown that hiPSC-EVs can inhibit primary cardiac and endothelial cells death [13, 15], we analyzed if hiPSC-EVs may have the protective effect on CB-HSPCs treated with staurosporine, as known inducer of apoptotic cell death [25]. Indeed, 2 h incubation with hiPSC-EVs significantly increased cell viability assessed by Anx V staining, following staurosporine treatment (Fig. 3C, D). In addition, we also observed pro-survival effect of hiPSC-EVs that enhanced viability of cells untreated with staurosporine (Fig. 3C, D). In conclusion, we demonstrated that hiPSC-EVs exhibit cytoprotective and pro-survival influence on CB-HSPCs.

### hiPSC-EVs enhance the chemotactic activity of CB-HSPCs in CXCR-independent manner

**Fig. 4:**
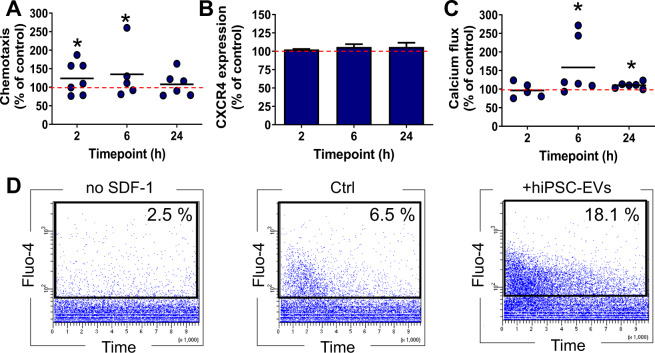
Influence of hiPSC-EVs on the chemotactic activity of CB-HSPCs. Prior experiment, cells were expanded for 7 days in the control medium without hiPSC-EVs and were subsequently treated with hiPSC-EVs for 2, 6, or 24 h. **A** Flow cytometry assessment of cell chemotaxis to SDF-1. Data are presented as a percentage of transmigrated hiPSC-EVs-treated cells compared to the control (cells untreated with hiPSC-EVs) in individual experimental repetitions (*N* = 7). Black lines represent the mean value, whereas the red line indicates the level of the control (100%). *p < 0.05 for control vs. hiPSC-EVs-treated cells, two-tailed Student’s *t*-test. **B** CXCR4 expression on the surface of CB-HSPCs after hiPSC-EVs treatment. Data were calculated as a percentage of the expression of the control (cells untreated with hiPSC-EVs) and are presented as the mean ± SD (*N* = 3). The red line indicates the level of the control (100%). **C** Influence of hiPSC-EVs on SDF-1-stimulated calcium flux in CB-HSPCs. Quantitative analysis of hiPSC-EVs-treated cells positive for Fluo-4 after SDF-1 stimulation, calculated as a percentage of the level of control cells (untreated with hiPSC-EVs) in individual experimental repetitions (*N* = 6). Black lines represent the mean value, whereas the red line indicates the level of the control (100%). *p < 0.05 for control vs. hiPSC-EVs-treated cells, two-tailed Student’s *t*-test. **D** Representative dot plots demonstrate the kinetics of changes in Fluo-4 signals from the baseline (no SDF-1; left plot), control (middle plot), and hiPSC-EVs-stimulated cells (right plot). The numbers on the plots represent the percentage of Fluo-4^+^ cells (present in black gates).

We tested whether hiPSC-EVs influence chemotactic response of CB-HSPCs to a crucial chemoattractant SDF-1, as it may determine efficiency of their homing into BM after the transplantation [26]. Indeed, hiPSC-EVs modulated CB-HSPCs response to chemotactic stimuli in a time-dependent manner, with significant increase following 2 and 6 h incubation with vesicles (Fig. 4A). Surprisingly, this effect did not correlated with the changes in expression of SDF-1 receptor CXCR4 on CB-HSPCs (Fig. 4B), which indicates other mechanisms underlying the hiPSC-EVs regulation of cell response to chemoattractant.

### Intracellular calcium flux increases in CB-HSPCs upon hiPSC-EVs treatment

As calcium flux as one of the signals involved in the initialization of hematopoietic cell SDF-1-driven motility [27], we incubated CB-HSPCs with hiPSC-EVs, loaded them with intracellular calcium concentration indicator Fluo-4 and immediately monitored fluorescent signal after exposition to SDF-1 (Fig. 4C, D). The results indicate that hiPSC-EVs increase the number of CB-HSPCs that responded to SDF-1 with calcium flux, with the highest effect observed after 6 h of treatment with vesicles (Fig. 4C, D). Collectively, these data indicate that hiPSC-EVs might promote SDF-1-stimulated calcium flux in CB-HSPCs, which may be partially responsible for the increase in their chemotactic activity.

### hiPSC-EVs promote CB-HSPCs adhesion to model elements of hematopoietic niche

**Fig. 5:**
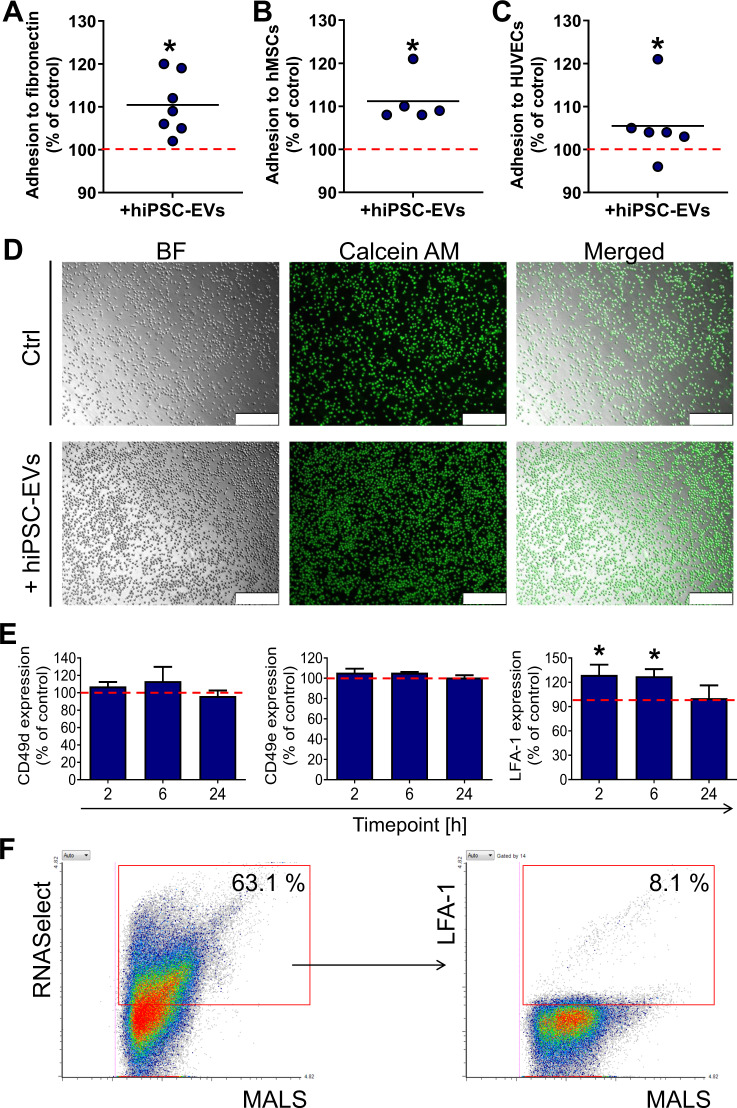
Effect of hiPSC-EVs on the adhesion capability of CB-HSPCs. Prior experiment, CB-HSPCs were expanded for 7 days in the control medium without hiPSC-EVs and were subsequently treated with hiPSC-EVs for 2 h. Next, cells were stained with calcein AM and seeded onto 96-well plates coated with fibronectin (**A**) or covered with the monolayer of hMSCs (**B**), and HUVECs (**C**). After the incubation, unbound cells were washed out, and fluorescence from the wells was measured by a plate reader. Data on the graphs present the averaged fluorescent signal from attached calcein AM-stained CD34^+^ cells calculated as a percentage of the control (cells untreated with hiPSC-EVs) in individual experimental repetitions (*N* = 7). Black lines represent the mean value, whereas the red line indicates the level of the control (100%). *p < 0.05 for control vs. hiPSC-EVs-treated cells, two-tailed Student’s *t*-test. **D** Representative images of calcein AM-labeled CB-HSPCs attached to fibronectin captured in brightfield (BF) and green fluorescence channel by Leica DMI6000B fluorescent microscope with 10× objective magnification. Scale bars indicate 250 µm. **E** Expression of adhesive molecules on CB-HSPCs treated with hiPSC-EVs for 2, 6, or 24 h. After the incubation, cells were stained with fluorescent-conjugated antibodies against CD49d, CD49e, and LFA-1 and acquired by flow cytometer. Data were calculated as a percentage of the median fluorescent intensity for the control (cells untreated with hiPSC-EVs) and is presented as the mean ± SD (*N* = 3). The red line indicates the level of the control (100%). *p < 0.05 for control vs. hiPSC-EVs-treated cells, two-tailed Student’s *t*-test. **F** Analysis of LFA-1 expression on hiPSC-EVs. Representative dot plots of hiPSC-EVs stained with RNASelect dye and anti-LFA-1 fluorescent antibody. The percentage of objects positive for the analyzed marker is shown in the red gate. MALS-medium angle light scatter parameter corresponds to the relative size of analyzed particles. In gating strategy, LFA-1 expression was analyzed only on hiPSC-EVs positive for RNASelect.

The ability of transplanted HSPCs to adhere to the endothelium and stromal cells is a crucial step for homing and engraftment into the BM [28]. Therefore, we tested whether hiPSC-EVs influence interaction of CB-HSPCs to model elements of hematopoietic niche. Indeed, 2 h incubation with hiPSC-EVs significantly increased CB-HSPCs adhesion to fibronectin as exemplary extracellular matrix protein (Fig. 5A, D), as well as to hMSCs (Fig. 5B) and HUVECs (Fig. 5C). However, there was no changes in the expression of surface antigens involved in hematopoietic cell adhesion [29], including CD49d (ligand for vascular cell adhesion molecule 1 and CD49e (ligand for fibronectin) [30]. On the other hand, the level of LFA-1 known as ligand for intercellular adhesion molecule 1 [31] was transiently increased in CB-HSPCs incubated with hiPSC-EVs (Fig. 5E). In addition, we verified the presence of LFA-1 on hiPSC-EVs, indicating that 8.1 ± 0.2% of RNA^+^ hiPSC-EVs possessed the LFA-1 marker (Fig. 5F), which indicates the possibility of LFA-1 transfer to CB-HSPCs upon hiPSC-EVs internalization.

In conclusion, hiPSC-EVs improve adhesion of CB-HSPCs to model elements of hematopoietic niche and the mechanism of this phenomenon might be partially related to the increased expression of LFA-1 on the surface of these cells.

### hiPSC-EVs modulate the expression of hematopoiesis-related genes in CB-HSPCs

**Fig. 6:**
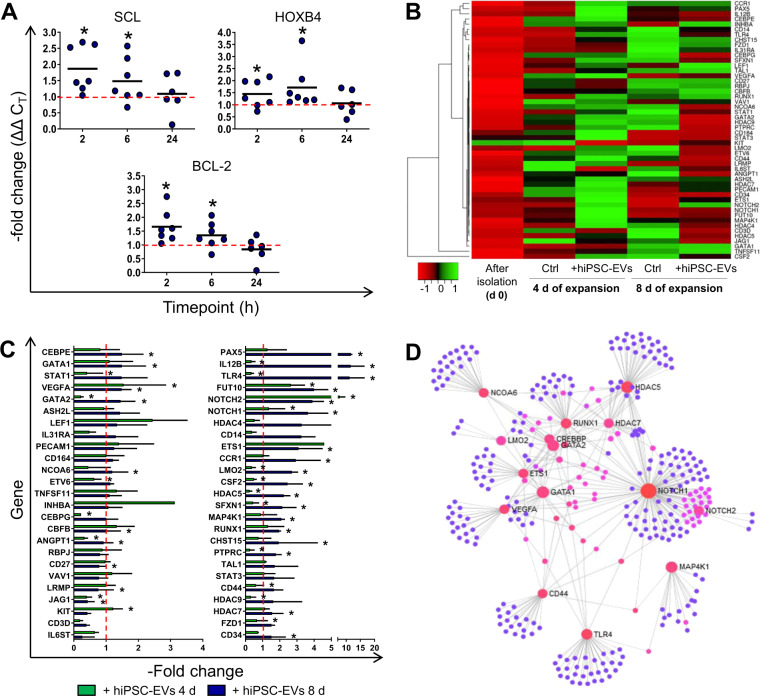
Influence of hiPSC-EVs on transcriptome changes in ex vivo expanded CB-HSPCs. **A** Influence of short-term treatment with hiPSC-EVs on gene expression in CB-HSPCs. Prior experiment, cells were expanded for 7 days in the control medium without hiPSC-EVs and were subsequently treated with hiPSC-EVs for 2, 6, or 24 h. The real-time PCR analysis of hematopoietic (*SCL* and *HOXB4*) and anti-apoptotic (*BCL-2*) mRNA levels in cells treated with hiPSC-EVs are presented as a –fold change compared to the level of the control (cells untreated with hiPSC-EVs) in individual experimental repetitions (*N* = 7). Black lines represent the mean value, whereas the red line indicates the level of control (expressed as 1). *p < 0.05 for control vs. hiPSC-EVs-treated cells, two-tailed Student’s *t*-test. **B**–**D** The effect of hiPSC-EVs on gene expression in cells during ex vivo expansion. CB-HSPCs were cultured in the control medium (Ctrl) or hiPSC-EVs medium (+hiPSC-EVs). On days 4 and 8 of the expansion, cells were harvested and analyzed with Hematopoiesis RT^2^ Profiler PCR Array. Freshly isolated cells (day 0) were also used in the analysis. **B** Heatmap representation of the relative mean expression of analyzed genes in subsequent sample types (*N* = 3) from the lowest (red) to the highest (green), based on the Row *Z*-Score parameter generated by Heatmapper Software. **C** Quantitative gene expression in cells expanded for 4 or 8 days in hiPSC-EVs medium. Data were calculated as a –fold change compared to the control (cells expanded in medium without hiPSC-EVs) and are presented as the mean ± SD (*N* = 3). The red line indicates the level of the control (expressed as 1). *p < 0.05 for control vs. hiPSC-EVs-treated cells, two-tailed Student’s *t*-test. **D** Analysis of gene interaction was performed by NetworkAnalyst Software implementing STRING Interactome database. Only genes upregulated on day 4 of expansion in hiPSC-EVs medium (–fold change >1 comparing to the control) were used in the analysis. Each analyzed gene is presented as a pink-orange sphere, and gray lines indicate the evidence of interaction and small blue spheres represent other interacting genes found by the software.

First, we tested whether short-term treatment with hiPSC-EVs may affect expression of selected genes in CB-HSPCs. We observed transient upregulation of the hematopoietic genes *SCL* and *HOXB4*, as well as anti-apoptotic *BCL-2* gene, following 2 and 6 h treatment with hiPSC-EVs (Fig. 6A). Next, using hematopoiesis PCR arrays we profiled expression of mRNA for several genes (Supplementary Table 5) potentially involved in the regulation of hematopoietic functions, in CB-HSPCs expanded for 4 or 8 days in the medium containing hiPSC-EVs. The results revealed a distinct pattern of mRNA expression for freshly isolated and expanded cells (Fig. 6B), with upregulation of most genes in expanded CB-HSPCs, when compared to freshly purified. The addition of hiPSC-EVs substantially influenced the level of several analyzed transcripts (Fig. 6B, C). In particular, upon hiPSC-EVs treatment 24 genes were significantly upregulated and 4 genes were downregulated on day 4, whereas on day 8 only 5 genes were upregulated and 16 downregulated (Fig. 6C and Supplementary Table 5).

Among genes upregulated following hiPSC-EVs addition, there was, e.g., *PAX5*, *NOTCH1* and *NOTCH2*, *FUT10*, *HDAC5* and *HDAC7*, as well as *RUNX1*, *GATA-1*, and *GATA-2* transcription factors. Bioinformatic analyses with the NetworkAnalyst tool implementing the STRING Interactome database showed a network of mutual, molecular interactions between particular genes and their protein products, whose expression was altered in cells expanded in the medium containing hiPSC-EVs (Fig. 6D). In summary, we demonstrated that both short-term and long-term treatment of CB-HSPCs with hiPSC-EVs influence the expression of selected genes associated with the control of hematopoietic cell function.

### hiPSC-EVs influence intracellular signaling pathways in CB-HSPCs

**Fig. 7:**
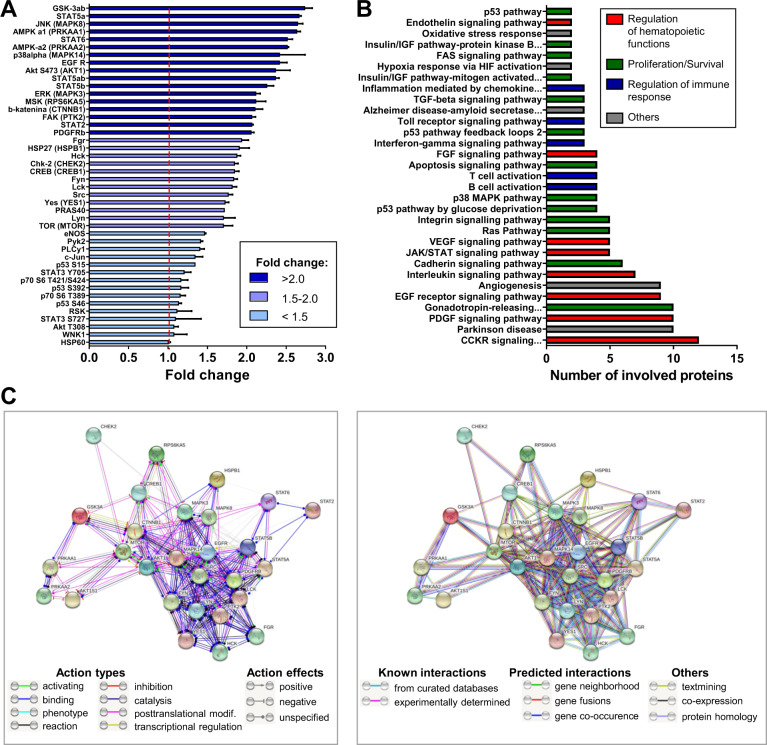
Semiquantitative profiling of kinases phosphorylation in hiPSC-EVs-treated CB-HSPCs. Prior experiment, cells were expanded for 7 days in the control medium without hiPSC-EVs and were subsequently treated with hiPSC-EVs for 2 h. Next, cell lysates were subjected to the assessment of protein phosphorylation by the Proteome Profiler Human Phospho-Kinase Array Kit. **A** Relative levels of protein phosphorylation on indicated amino acids are presented as a –fold change compared to the control (cells untreated with hiPSC-EVs). Data are presented as the mean ± SD (*N* = 2). The red line indicates the level of the control (expressed as 1). When few phosphorylation sites were analyzed within the same protein, a particular amino acid site was indicated. Alternative protein names are included in the brackets. The color coding of the graph bars corresponds to the phosphorylation level ranges as indicated on the graph legend. **B** PANTHER Pathway Overrepresentation Test. Only proteins with phosphorylation –fold change >1.5 were used in the analysis. The graph presents molecular pathways (PANTHER Pathways) that involve the indicated number of analyzed protein subsets. The color coding of the graph bars, indicated on the graph legend, corresponds to the subjective classification of pathways according to their predominant function in hematopoietic cells. **C** STRING analysis of the interaction between proteins (presented as spheres) used in the PANTHER analysis was performed in two modes. Left panel: “Molecular action” mode analysis. Line colors between proteins indicate the predicted mode of action, including action types or action effects. Right panel: “Evidence” mode analysis. Line colors between proteins indicate the type of interaction evidence, including known interactions, predicted interactions, or others.

In order to evaluate whether hiPSC-EVs might change the activity of intracellular signaling pathways regulating cell fate, we assessed the changes in the phosphorylation of several proteins, including kinases controlling intracellular signal transduction. We observed that the 2 h incubation of CB-HSPCs with hiPSC-EVs triggers global changes in the level of protein phosphorylation (Fig. 7A and Supplementary Table 6). Among the kinases with upregulated phosphorylation, we found for example those involved in signal transducer and activator of transcription (STAT) pathway (Fig. 7A and Supplementary Table 6), including STAT2, STAT5ab, and STAT6. Moreover, phosphorylation of GSK3-ab on serine (S) 21/S9, known to cause its inhibition that may lead to the promotion of cell survival and proliferation [32], was also elevated. We also observed stronger phosphorylation of Akt and AMPK kinases in CB-HSPCs incubated with hiPSC-EVs, which may lead to energy preservation and cell survival [33, 34], as well as an upregulation of mitogen-activated protein kinase (MAPK) pathways through the hyper-phosphorylation of three MAP kinases involved in the regulation of hematopoiesis, including ERK, JNK, and p38alpha [35]. The activity of FAK kinase was also upregulated.

In addition, PANTHER Classification System divided proteins with upregulated phosphorylation upon cell treatment with hiPSC-EVs (fold change >1.5 as compared to the control) into 31 distinct biological pathways, which may be classified as pathways involved in the regulation of hematopoietic functions, cell proliferation/survival, and regulation of the immune response (Fig. 7B and Supplementary Table 7). These proteins were also clustered using the STRING tool, which showed their strong, multidirectional interactions (Fig. 7C). Taking together, we revealed that hiPSC-EVs may change the activation of several intracellular signaling pathways in CB-HSPCs, which might influence their hematopoietic functions.

### hiPSC-EVs improve homing and engraftment of CB-HSPCs in vivo

**Fig. 8:**
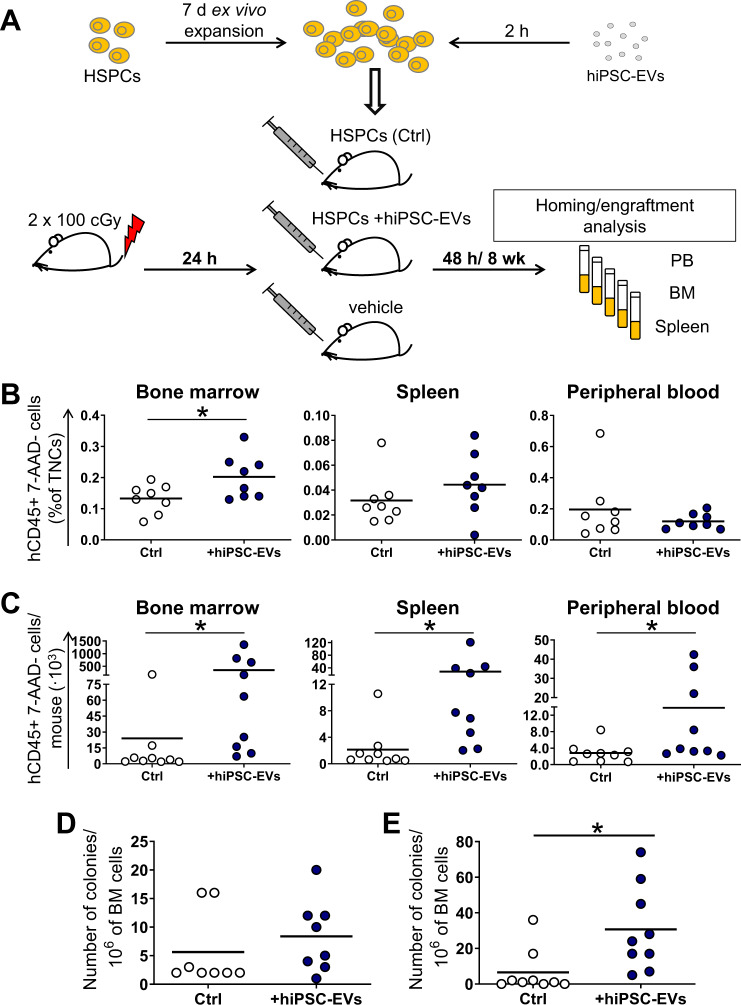
Analysis of hiPSC-EVs influence on homing and engraftment of CB-HSPCs in vivo. **A** Schematic layout of the experiment. Prior experiment, cells were expanded for 7 days in the control medium without hiPSC-EVs and were subsequently treated with hiPSC-EVs for 2 h. Untreated cells served as the control (Ctrl). Next, cells were transplanted into γ-irradiated NOD/SCID mice. After the transplantation, mice were sacrificed at 48 h (homing analysis; *N* = 8) or 8 weeks (engraftment analysis; *N* = 9), and their tissues were harvested. The number of live (7-AAD-) human hematopoietic (hCD45^+^) cells in BM, spleen, and PB isolated from mice 48 h (**B**) or 8 weeks (**C**) after the transplantation was analyzed by BD LSRFortessa flow cytometer and expressed as a percentage of the total nucleated cells (TNCs) or as the number of cells/mice, respectively. Each dot represents the results for individual animals. Black lines represent the mean value. *p < 0.05 for control vs. hiPSC-EVs-treated cells. **D**, **E** Colony-forming cell assay on BM cells isolated from transplanted mice. Data on the graph present the number of human hematopoietic colonies grown from the 10^6^ of BM cells isolated from individual mice 48 h (**D**) or 8 weeks (**E**) after the transplantation. The black line represents the mean value. *p < 0.05 for control vs. hiPSC-EVs-treated cells, two-tailed Student’s *t*-test.

Based on results in vitro, we also tested whether hiPSC-EVs might stimulate the homing and engraftment of CB cells in vivo. Hence, we transplanted expanded CB-HSPCs, pre-incubated for 2 h with hiPSC-EVs, to a sublethally γ-irradiated NOD/SCID mice (Fig. 8A). Forty-eight hours after the transplantation, we observed significant increase in the number of human hematopoietic (hCD45^+^) cells in the BM of mice transplanted with CB-HSPCs treated with hiPSC-EVs (Fig. 8B). In addition, the number of hCD45^+^ cells was also slightly higher in spleens but lower in PB isolated from mice transplanted with hiPSC-EVs-treated cells (Fig. 8B; *p* > 0.05). These results indicate improvement of CB-HSPCs homing following migration from PB to hematopoietic niches.

Next, we also analyzed mice tissues 8 weeks after the transplantation of CB-HSPCs, demonstrating improved engraftment following hiPSC-EVs treatment, as the number of hCD45^+^ cells was significantly higher in murine BM (14.5-fold), spleens (13-fold), and PB (5-fold) in mice transplanted with CB-HSPCs incubated with hiPSC-EVs (Fig. 8C). Moreover, CFC assay 8 weeks post transplantation revealed that the number of human hematopoietic colonies was higher for BM of mice transplanted with hiPSC-EVs-treated CB-HSPCs (Fig. 8E). Similar tendency was also observed for CFC assay on BM isolated 48 h post transplantation (Fig. 8D; *p* > 0.05). In conclusion, hiPSC-EVs may enhance the migration of transplanted CB-HSPCs from PB into the hematopoietic niches, which increases homing and engraftment capacity of these cells.

## Discussion

Since the number of CB-HSPCs present in a single CB unit significantly limits its efficient clinical application in adult patients [36], several approaches have been proposed for ex vivo “priming” of CB-HSPCs to enhance their potential and reduce the risk of graft failure after CB transplantation. Among the proposed approaches, the use of EVs rises new possibilities and interest in hematology [37]. Recently, few groups have published data showing that EVs isolated from BM-derived hMSCs and osteoblasts may impact on CB cells enhancing their therapeutic efficacy [9, 38, 39]. However, as the biological content of released EVs strongly depends on the type of their parental cells [40], it remains unknown whether EVs isolated from more developmentally primitive SCs types may be also beneficial or act even more efficiently on CB-HSPCs and modulate their functions. Indeed, Ratajczak et al. [41] showed that EVs released by embryonic SCs may stimulate the expansion of hematopoietic progenitors and facilitate their survival. Moreover, we showed in our previous studies that hiPSCs-EVs significantly impact on other target cells improving their pro-regenerative functions [13]. Thus, we decided to investigate whether hiPSCs-EVs may also support hematopoietic functions of CB-HSPCs, which has never been studied before.

First, while we confirmed the submicron size of isolated hiPSC-EVs, possessing differential content of exosomal, ectosomal, and other surface markers of their parental cells [13]. Subsequently, we demonstrated that hiPSCs-EVs may enter CB-HSPCs, influencing their functions in vitro and in vivo. Although there was no effect of hiPSC-EVs on CB-HSPCs ex vivo expansion, we observed a significant increase in cell metabolic activity, hematopoietic differentiation, and clonogenicity, which correlated with global activation of several intracellular pathways, including, Akt/mTOR, STAT, or MAPK, which are known to be involved in the regulation of hematopoietic cell behavior [35, 42]. In addition, these results could be supported by our other observations including the increase in the expression of hematopoietic genes and the activation of kinases involved, e.g., in TGF-β signaling, which might promote differentiation of HSPCs as described before [43].

In our experiments, we also demonstrated that hiPSC-EVs might trigger pro-survival signals and possess cytoprotective effects on CB-HSPCs, which is consistent with increased anti-apoptotic *BCL-2* gene expression and pro-survival kinase signaling. In addition, the results are supported by our previous studies [13, 15] and others, showing protective activity of iPS-EVs in several disease models, including myocardial infarction [14] or lung epithelial wound-healing [44].

We also revealed an increase in the chemotactic activity of the CB-HSPCs following hiPSC-EVs treatment, which corresponded to the increased SDF-1-triggered calcium flux, shown to be an early event involved in the activation of chemotaxis [45], but was not correlated with changes in CXCR4 expression, suggesting the role of distinct mechanisms. It has been previously shown that the fusion of EVs with cell membrane may change its properties, influencing accessibility to external stimuli, as, e.g., was described for EVs released by the bacteria *Porphyromonas gingivalis* that affected cell membrane fluidity of neutrophils [46]. We hypothesize that the binding of hiPSC-EVs might potentially change the localization of CXCR4 within lipid rafts, affecting their response to chemotactic signaling [47]. Nevertheless, this hypothesis must be further examined. In addition, our results demonstrated that hiPSC-EVs activated several intracellular pathways in CB-HSPCs including FAK signaling, involved in the migratory capacity of cells [48]. As cell migration requires the adhesion to the ECM and interaction with neighboring cells, we also showed the positive effect of hiPSC-EVs on the adhesion of CB-HSPCs to model elements of hematopoietic niche, which corresponded to the activation of integrin and cadherin signaling. In addition, we observed the upregulation of LFA-1 protein on the surface of cells treated with hiPSC-EVs, which could contribute to the enhancement of their adhesive interactions [49].

Despite significant progress in the field, the exact mechanisms of EV interactions with target cells remain unclear and little is known about their mode of action on the HSPCs. It is postulated that EVs may fuse with the cell membrane of target cells and the release their bioactive cargo, predominantly mRNA and miRNA, which impacts cell behavior [50]. Indeed, profiling of EVs derived from osteoblasts and BM-hMSCs has shown the presence of several miRNAs involved in the regulation of hematopoietic development [9, 38, 39]. In addition, our previous study [13] demonstrated that hiPSCs-EVs are enriched in miR-20a-5p, miR-106a, miR-125a, and miR-130a, which were shown to influence hematopoietic cell fate, including regulation of HSPCs proliferation and differentiation [51, 52].

We also evaluated the effect of hiPSC-EVs on the expression of genes related to the different cellular and hematopoietic functions. Our results revealed substantial changes in the mRNA profile of CB-HSPCs, with, e.g., an increase in the level of the *PAX5* regulating B-cell lineage development [53], *NOTCH1* and *NOTCH2* involved in the modulation of hematopoietic cell function [54], *GATA-1* and *GATA-2* controlling erythropoiesis and megakaryopoiesis [55], as well as *RUNX1* playing a key role in lymphopoiesis and differentiation towards megakaryocytes [56].

Apart from changes in the mRNA level, we have also shown that short incubation with hiPSC-EVs alters the activity of several intracellular pathways in CB-HSPCs, through affecting phosphorylation of kinases, including those contributing to hematopoiesis and hematopoietic cell fate regulation. As an example, there was upregulated phosphorylation of proteins involved in PDGF signaling, previously shown to elevate the engraftment of these cells in NOD/SCID mice [57], EGFR and FGF signaling that may affect HSPCs survival and recovery [58, 59], as well as insulin/IGF and TGF-β signaling pathways controlling proliferation and differentiation of HSPCs [43, 60].

Importantly, we showed that expanded CB-HSPCs cells pre-incubated with hiPSC-EVs possessed considerably higher capacity to home and engraft into hematopoietic niches in vivo, which not only confirms possible utility of these EVs in the context of functional improvement of CB cells, but also indicates potential advantage of hiPSC-EVs when compared to other EV sources. For example, contrary to our results, Morhayim et al. [39] showed that treatment of CB-derived CD34^+^ with osteoblast-derived EVs had no effect on BM chimerism following transplantation.

In conclusion, our findings for the first time demonstrated that hiPSC-EVs might improve several functions of CB-HSPCs important for their homing and hematopoietic activity following transplantation. We also provided important insight into the mechanisms of the biological activity of hiPSC-EVs and demonstrated their potential feasibility in clinical applications. Based on these data, the use of hiPSC-EVs may be a novel, promising strategy to increase the hematopoietic potential of CB-HSPCs in ex vivo pre-conditioning protocols, and thus contribute to improving the applicability of this graft material in clinical practice.

## Supplementary information

Supplementary Information

Supplementary Movie 1
