# Supplementary material for: Extracellular vesicles from human iPSCs enhance reconstitution capacity of cord blood-derived hematopoietic stem and progenitor cells
Source: Leukemia. 2021 Jun 17;35(10):2964–77. doi: 10.1038/s41375-021-01325-y (PMC8478657; doi:10.1038/s41375-021-01325-y)
Supplement: Supplementary file 1 — Supplementary Information [file 41375_2021_1325_MOESM1_ESM.docx]

**SUPPLEMENTARY INFORMATION**

**Supplementary Methods**

**Cord blood processing and cell isolation**

To remove red blood cells, CB units were treated twice with 1× BD Pharm Lyse buffer (BD Bioscience, San Jose, CA, USA) at room temperature (25 °C; RT) for 15 min and washed in phosphate-buffered saline without Ca^2+^ and Mg^2+^ (PBS; HyClone GE Healthcare, Chicago, IL, USA). Initial purification of the remaining total nucleated cells (TNCs) was performed using magnetic-activated cell sorting (MACS). Before separation, TNCs were resuspended in MACS Buffer (autoMACS Rinsing Solution supplemented with MACS BSA Stock Solution, according to manufacturer’s protocol; Miltenyi Biotec, Bergisch Gladbach, Germany) containing 2% of FBS (Sigma-Aldrich). Next, TNCs were stained with a CD34 Microbead kit (Miltenyi Biotec), according to the manufacturer’s protocol. Positive cell fractions were separated by AutoMACS Pro system (Miltenyi Biotec) and subsequently re-purified by fluorescence-activated cell sorter (FACS) BD FACSAria III (BD Bioscience). Prior to FACS, cells were fluorescently stained for 30 min at 4°C using monoclonal antibodies (all from BD Bioscience) against the following antigens: FITC-conjugated lineage (Lin) markers (CD2 (clone RPA-2.10), CD3 (clone UCHT1), CD14 (clone M5E2), CD16 (clone 3G8), CD19 (clone HIB19), CD24 (clone ML5), CD56 (clone NCAM16.2), CD66b (clone G10F5), and CD235a (clone GA-R2)), PE-conjugated CD45 (clone HI30), and APC-conjugated CD34 (clone 581). After washing with PBS, the CD45^dim^Lin^-^CD34^+^ fraction highly enriched in HSPCs was sorted for further analyses.

**Isolation of hiPSC-EVs**

hiPSC-EVs were isolated by sequential ultracentrifugation of the conditioned medium (CM) collected from hiPSCs, as previously described. Briefly, CM from semi-confluent hiPSC cell culture grown in Essential 8 medium (Thermo Fisher Scientific, Waltham, MA, USA) was first centrifuged at 2000 *g* for 20 min at 4 °C to remove cell debris and apoptotic bodies. Next, supernatants were collected and spun down by ultracentrifugation at 100 000 *g* for 1 h at 4 °C using an Optima XPN-90 ultracentrifuge and type 50.2 Ti fixed-angle rotor (both from Beckman Coulter, Brea, CA, USA). Pellets were then washed in 0.2 µl-filtered PBS without Ca^2+^ and Mg^2+^ (Lonza) and ultracentrifuged again with the same parameters.

**Generation of copGFP-expressing hiPSC cell line**

copGFP-expressing lentiviral vector was created by deleting an 880-bp DNA fragment from the PMIRH1-1-PA plasmid (System Biosciences, Palo Alto, CA, USA) using Cla I and Not I restriction enzymes (Eurx, Gdansk, Poland), then blunting DNA ends with the Quick Blunting Kit (New England Biolabs, Ipswich, MA, USA) and re-ligating with T4 DNA ligase (Thermo Fisher Scientific). The copGFP-expression vector was used for lentiviral vector production by co-transfection of the HEK293T/17 packaging cell line (ATCC-CRL-11260; LGC Standards) with psPAX2 and pMD2G packaging plasmids (#12260 and #12259, respectively; Addgene, Watertown, MA, USA) using Lipofectamine2000 (Thermo Fisher Scientific) as a transfection agent. Cell supernatant was filtered through 0.2 mm pores PVDF filters (Merck Millipore, Burlington, MA, USA) and was titrated on HEK293T cells. hiPSCs were transduced with the lentiviral infectious particles at an MOI of 2 in Essential 8 medium with the addition of 2 mg/ml of polybrene (Merck Millipore). After 9 d of expansion, the copGFP-positive cells were sorted using the FACS Aria III cell sorter.

**hiPSC-EVs characterization**

*Nanoparticle tracking analysis (NTA)*

The size distribution of isolated hiPSC-EVs was measured by a NanoSight NS300 analyzer (Malvern Pananalytical, Malvern, UK). For the measurement, hiPSC-EVs samples obtained after the ultracentrifugation was diluted in 0.2 µm-filtered DPBS (Lonza) to the final concentration of 2 µg of protein/ml. Three repetitions of each sample tracking were performed with camera level 13 and the threshold parameter set on 3. Mean particle concentration and size distribution were calculated by NTA Software (Malvern Pananalytical) based on the data obtained from samples prepared from three independent hiPSC-EVs isolations.

*Atomic Force Microscopy (AFM)*

AFM analysis was performed according to the previously described protocol [17, 19]. Briefly, the hiPSC-EVs droplet was placed on a mica surface, incubated for 20 min at RT, and washed carefully with PBS. The BioScope Catalyst AFM system (Brüker, Billerica, MA, USA) was used to capture the 2D and 3D images of EVs in PeakForce Tapping mode using a silicon nitride probe with a tip radius of 2 nm.

*Flow cytometry*

hiPSC-EVs were stained with RNASelect dye (Thermo Fisher Scientific) and one of the following mouse monoclonal antibodies conjugated with allophycocyanin (APC): anti-CD9 (clone MEM-61), anti-CD63 (clone MEM-259; both from Thermo Fisher Scientific), anti-CD34 (clone 581), anti-CD45 (clone HI30), anti-CD81 (clone 5A6), anti-CD90 (clone 5E10), anti-CD105 (clone 43A3), anti-Tra-1-60 (clone TRA-1-60R), anti-SSEA-4 (clone MC-813-70), anti-LFA-1 (clone m24; all from BioLegend, San Diego, CA, USA), anti-KDR (clone 89106; R&D Systems), or appropriate isotype-match controls. Prior to staining, RNASelect dye and all antibodies were suspended in 0.2 µm-filtered DPBS (Lonza) and centrifuged at 21 000 *g* for 20 min at 4 °C to remove potential debris and protein aggregates. Next, supernatants were taken to fresh Eppendorf tubes, and then hiPSC-EVs samples were added for 30 min at 4 °C. The analysis of stained hiPSC-EVs was performed with an Apogee A50-Micro flow cytometer (Apogee Flow Systems, Hemel Hempstead, UK) dedicated for the characterization of small particles. Prior to the analysis, the system was calibrated using Apogee calibration beads (#1493; Apogee Flow Systems). The percentage of gated positive events was calculated by Histogram software (Apogee Flow Systems). To ensure the reliability of the obtained data, appropriate isotype controls were included in the gating strategy. Additionally, samples containing only reagents without hiPSC-EVs were also acquired to verify the absence of potential background particles (data not shown).

*Western blotting*

10 μg of denatured protein lysates from hiPSC-EVs and their parental cells were loaded onto Mini-PROTEAN TGX Precast Gels (Bio-Rad, Hercules, CA, USA) and separated by electrophoresis. PageRuler Prestained Protein Ladder (Thermo Fisher Scientific) was also loaded as a size marker. Next, the transfer of samples into PVDF membranes was performed with Trans-Blot Turbo RTA Mini PVDF Transfer Kit (Bio-Rad). Subsequently, membranes were blocked for 1 h with 1% BSA in TBST buffer and then incubated with primary antibodies against CD9, CD63 (both mouse monoclonal IgG, Thermo Fisher Scientific), syntenin, and calnexin (both goat polyclonal IgG; Thermo Fisher Scientific). Mouse monoclonal IgG antibody (Thermo Fisher Scientific) was used to detect β-actin as a control protein. The level of protein expression was detected with horseradish peroxidase (HRP)-conjugated rabbit anti-goat IgG or goat anti-mouse IgG secondary antibodies (both from Thermo Fisher Scientific). All antibodies were used in the dilutions as recommended in the manufacturer’s protocols. Prior to the detection of protein signals with the Gel Doc XR+ Gel Documentation System (Bio-Rad), Luminata Crescendo Western HRP Substrate (Merck Millipore) was added onto the membranes to develop the signal. Luminescent signals were captured and analyzed using Image Lab software (Bio-Rad).

**Fluorescence microscopy**

To visualize the entrance of hiPSC-EVs into CB-HSPCs, expanded cells were seeded for 2 h onto WillCo-dish glass bottom 3.5 cm dishes (WillCo Wells, Amsterdam, Netherlands) covered with fibronectin (50 µg/ml; Sigma-Aldrich) to facilitate cell attachment. Next, copGFP+ hiPSC-EVs were added into the culture medium with a concentration of 2 µg/ml. After 2 h of co-incubation, unbound hiPSC-EVs were washed out with fresh medium, and cells were subjected to the fluorescence microscopy observations. Prior to the analysis, Hoechst 33342 (Hoe) fluorescent dye (1 µg/ml; BD Bioscience) was added into the cells to stain the nuclei. Image acquisition was performed with a Leica DMI6000B microscope (DMI7000 version; Leica Microsystems, Wetzlar, Germany) equipped with the differential interference contrast module. Images were registered with 100x NA-1.47 oil immersion objective at 37 °C with 5% CO_2_ using a 14-bit Hamamatsu 9100–02 EM-CCD camera controlled by the Leica Application Suite Advanced Fluorescence software. For Hoe and copGFP visualization, the “A4” and “GFP” filter-cubes were used, respectively. LAS-AF deconvolution software (Leica Microsystems) was used for image processing.

**Metabolic activity assessment**

After 7 d of the *ex vivo* expansion of CB-HSPCs, 5×10^4^ of cells were seeded in triplicate onto 96-well white plate (Perkin Elmer, Waltham, MA, USA) in medium with or without hiPSC-EVs. Subsequently, cells were cultured for the next 2 h, 24 h, and 48 h. After those times, the concentration of ATP produced by the cells was measured by the ATPLite Luminescence Assay System (Perkin Elmer), according to the manufacturer’s protocol. Luminescent signals from individual wells were acquired using the Infinite M200 Pro analyzer and i-control Software (Tecan, Männedorf, Switzerland). ATP concentration was calculated based on the calibration curve obtained for provided ATP standards.

**Analysis of CB-HSPCs phenotype kinetics**

At the indicated time points of the *ex vivo* expansion (4, 6, 11 and 14 d), CB-HSPCs cultured in the control medium or hiPSC-EVs medium were harvested and stained in MACS Buffer containing 2% of FBS for 30 min at 4 °C with FITC-conjugated antibodies against Lin markers, including: T-cells (CD3; clone UCHT1), B-cells (CD19; clone HIB19), monocytes/macrophages (CD14; clone M5E2), granulocytes (CD66b; G10F5), and erythroid cells (CD235a; clone GA-R2); and APC-conjugated anti-CD34 antigen (all from BD Bioscience) defining hematopoietic stemness of the cells. After staining, cells were washed with PBS and resuspended in MACS Buffer containing 2% of FBS. Next, the analysis of antigen expression was performed with LSRFortessa flow cytometer and BD FACSDiva software version 8.1 (BD Bioscience).

**Colony-forming cell (CFC) assay**

On 4, 6, 11 and 14 d of the *ex vivo* expansion, CB-HSPCs cultured in the control medium or hiPSC-EVs medium were harvested, and 5×10^2^ cells were seeded onto 3.5 cm Petri dishes (Falcon) in a semi-liquid methylcellulose (Human Methylcellulose Enriched Media; R&D Systems, Minneapolis, MN, USA) containing SCF (50 ng/ml), GM-CSF, G-CSF, IL-3, IL-6 (all 20 ng/ml), and EPO (3 U/ml). After 14 d of culture, the following types of hematopoietic colonies were counted under the light microscope (Olympus IX81, Olympus Corp., Tokyo, Japan) based on morphology: burst forming unit-erythroid (BFU-E), colony-forming unit-granulocyte-macrophage (CFU-GM), and colony-forming unit-granulocyte, erythrocyte, macrophage, megakaryocyte (CFU-GEMM). Analogically, CFC assay was also performed on CB-HSPCs in short-term approach, where cells were first expanded for 7 d and subsequently treated with hiPSC-EVs for 2 h, 6 h, or 24 h.

**Apoptosis assay**

CB-HSPCs were incubated for 2 h in the control medium or hiPSC-EVs medium after 7 d of *ex vivo* expansion. Next, staurosporine (100 nM; Sigma-Aldrich) was added to the culture medium. Cells without the staurosporine addition served as a test control. After 24 h of further culture, cells were harvested and stained with Annexin V (Anx V) Apoptosis Detection Kit (BD Bioscience), according to manufacturer’s protocol, and analyzed by LSRFortessa flow cytometer. The percentage of live (Anx V- 7-aminoactinomycin D (7-AAD)-), early-apoptotic (Anx V+ 7-AAD-), late-apoptotic (Anx V+ 7-AAD+), and necrotic cells (Anx V- 7-AAD+) was analyzed by BD FACSDiva version 8.1 software.

**Chemotaxis assay**

CB-HSPCs were incubated for 2 h, 6 h, and 24 h in control medium or hiPSC-EVs medium after 7 d of *ex vivo* expansion. Next, cells were centrifuged (300 *g*, 5 min, RT) to remove unbound hiPSC-EVs and were resuspended in IMDM medium (Sigma-Aldrich) supplemented with 0.5% bovine serum albumin (BSA; Sigma-Aldrich). Chemotaxis assay was performed by seeding 1.5×10^5^ of cells onto transwell inserts with 5 µm pore diameter (Corning) placed in 24-well plate filled with IMDM medium supplemented with 0.5% BSA and containing SDF-1 (100 ng/ml; Peprotech, Rocky Hill, NJ, USA). Medium without the SDF-1 addition served as a negative control. Additionally, 3×10^4^ cells were also seeded as a quantity control onto wells without transwell inserts. After 3 h of incubation (37 °C, 5% CO_2_), transmigrated cells were harvested from the lower compartment of the wells and counted by LSRFortessa flow cytometer. The percentage of cell chemotaxis was calculated as the number of transmigrated cells x 20/ number of cells for the quantity control.

Before seeding on transwell inserts, a part of the cells was stained with PE-Cy7 anti- C-X-C motif chemokine receptor 4 (CXCR4) antibody (clone 12G5; BD Bioscience) and analyzed by flow cytometry, to evaluate the level of the expression of this receptor for SDF-1.

**Calcium flux**

CB-HSPCs (2×10^5^) were incubated for 2 h, 6 h, and 24 h in the control medium or hiPSC-EVs medium after 7 d of *ex vivo* expansion. Next, unbound hiPSC-EVs were washed out, and cells were resuspended in IMDM medium (Sigma-Aldrich) supplemented with 0.5% BSA and 1 µM Fluo-4 calcium indicator (Thermo Fisher Scientific). Cells were loaded with Fluo-4 for 30 min at 37 °C, washed twice in PBS and resuspended in IMDM with 0.5% BSA. Calcium flux was initiated by the addition of IMDM with 0.5% BSA containing 200 ng/ml SDF-1 into the cell suspension in 1:1 ratio (v/v) to the final concentration of SDF-1 100 ng/ml. Fluorescence signals from Fluo-4 were immediately measured by LSRFortessa flow cytometer in the FITC-channel. The signal acquired for cells before the addition of SDF-1 served as a baseline. Each sample was acquired for 100 s. Calcium flux was assessed based on the percentage of FITC-positive cells.

**Adhesion assay**

CB-HSPCs were incubated for 2 h in the control medium or hiPSC-EVs medium after 7 d of *ex vivo* expansion. Additionally, after 1.5 h of incubation with hiPSC-EVs, calcein AM (Vybrant Cell Adhesion Assay kit; Thermo Fisher Scientific) was added for 30 min to stain the cells, according to the manufacturer’s protocol. Cells were then washed with PBS, resuspended in StemSpan SFEM medium, and seeded in triplicate onto fibronectin-coated (50 µg/ml, 1 h in RT) black 96-well culture plates with a transparent bottom (Corning, Corning, NY, USA) in the number of 3×10^5^ cells/well. Subsequently, cells were allowed to attach to wells for 2.5 h at 37 °C, and then unbound cells were removed by plate whisking and double washing with PBS. Next, the averaged signal from attached calcein AM-stained CB-HSPCs was measured by Infinite M200 Pro analyzer (excitation 494 nm, emission 517 nm) and i-control Software (Tecan, Männedorf, Switzerland). Additionally, images of the attached cells were captured in brightfield and green fluorescence channels by a Leica DMI6000B microscope (Leica Microsystems). Analogously, an adhesion assay was also performed by seeding HSPCs treated with hiPSC-EVs onto the wells covered with the monolayer of hMSCs or HUVECs.

**Expression of adhesion markers**

CB-HSPCs were incubated for 2 h, 6 h, and 24 h in the control medium or hiPSC-EVs medium after 7 d of *ex vivo* expansion. Next, unbound hiPSC-EVs were washed out, and cells were resuspended in MACS Buffer containing 2% of FBS and stained for 30 min at 4 °C with the following mouse monoclonal antibodies (all from BioLegend): BV421-conjugated anti-CD49d (clone 9F10), PE-conjugated anti-CD49e (clone NKI-SAM-1), or APC-conjugated anti-LFA-1 (clone m24). Next, the analysis of antigen expression (assessed based on median fluorescent intensity (MFI)) was performed with LSRFortessa flow cytometer and BD FACSDiva software version 8.1.

**Gene expression**

Changes in the expression of hematopoietic-related genes in CB-HSPCs treated with hiPSC-EVs were assessed for cells shortly incubated or constantly expanded in hiPSC-EVs medium.

In the short-term treatment approach, cells were incubated for 2 h, 6 h, or 24 h in the control medium or hiPSC-EVs medium after 7 d of *ex vivo* expansion. Total mRNA was isolated by GeneMATRIX Universal RNA Isolation Kit (Eurx), and 300 ng of RNA was reverse transcribed with TaqMan Reverse Transcription reagents (Thermo Fisher Scientific), according to manufacturer protocols. The obtained cDNA was used for the detection of mRNA expression for *SCL*, *HOXB4,* and *BCL-2* genes. β2-microglobulin was used as an endogenous control. All primers were used at a final concentration of 5 µM (Genomed, Warsaw, Poland; sequences of used primers are listed in Supplementary Table 1). Quantitative real-time PCR (qPCR) was performed using SG qPCR Master Mix (Eurx) and 7500 Fast Real-Time PCR System (Thermo Fisher Scientific). Relative changes in gene expression were calculated with the comparative C_T_ (threshold cycle) method. The relative quantitative values of the target genes normalized to the endogenous control were expressed as 2^-ΔΔCT^ (fold change), where ΔC_T_ = (C_T_ of target genes) – (C_T_ of endogenous control), and ΔΔC_T_ = (ΔC_T_ of samples for target gene) – (ΔC_T_ of calibrator for the target gene). Data obtained for cells without hiPSC-EVs treatment (incubated in control medium) served as a calibrator.

In the long-term treatment approach, freshly isolated CB-HSPCs were cultured for 4 d or 8 d in the control medium or hiPSC-EVs medium. Cells just after the isolation (0 d) were also subjected to the analysis. Total mRNA was isolated and reverse transcribed as previously described, and the obtained cDNA was then analyzed with Hematopoiesis RT^2^ Profiler PCR Arrays (Qiagen, Hilden, Germany), according to manufacturer’s protocol. The qPCR reaction was conducted with the SYBR Green qPCR Master Mix (Thermo Fisher Scientific) and QuantStudio 6 Flex Real-Time PCR System (Thermo Fisher Scientific). The relative quantitative values of the analyzed genes (Supplementary Table 2) normalized to the endogenous control (β2-microglobulin) were expressed as 2^-ΔΔCT^ (fold change) using either freshly isolated cells or cells expanded in medium without hiPSC-EVs as a calibrator.

**Semiquantitative assessment of kinase activity**

CB-HSPCs were incubated for 2 h in the control medium or hiPSC-EVs medium after 7 d of *ex vivo* expansion. Then, unbound hiPSC-EVs were washed out. Next, the relative levels of the selected 43 protein phosphorylations (proteins are listed in Supplementary Table 3) in analyzed cells were evaluated by Proteome Profiler Human Phospho-Kinase Array Kit (R&D Systems). Experiments were followed according to the manufacturer’s protocol. Briefly, cells were lysed, and the obtained lysates were incubated overnight at 4 °C with arrays composed of nitrocellulose membranes containing duplicated spots of capture antibodies. After washing with a dedicated wash buffer, biotinylated detecting antibodies were added for 2 h at RT. Next, arrays were washed and incubated with streptavidin-conjugated horseradish peroxidase (HRP). Prior to the analysis, membranes were covered with the appropriate HRP substrate, and then chemiluminescent signals were detected by the MicroChemi analyzer (DNR Bio-Imaging Systems, Neve Yamin, Israel). Densitometric analyses of averaged pixel density from duplicate spots were performed by Quantity One Software (Bio-Rad).

The data were analyzed with PANTHER (Protein Analysis Through Evolutionary Relationships) Pathway Classification System version 13.1 (http://www.pantherdb.org/) in the Pathway Overrepresentation Test mode. Only proteins with phosphorylation-fold change > 1.5 as compared to control cells were taken into the analysis and assigned into PANTHER Pathways. Additionally, the STRING tool (Search Tool for the Retrieval of Interacting Genes/Proteins) ver. 10.5 (https://string-db.org/) was also used to perform the analysis of the interaction between proteins taken into the PANTHER analysis. Two analysis modes were utilized: ‘Molecular action’ indicating the predicted mode of action, or ‘Evidence’ indicating the type of interaction evidence.

***In vivo* BM reconstitution assay**

*Cell preparation and transplantation*

CB-HSPCs were incubated for 2 h in the control medium or hiPSC-EVs medium after 7 d of *ex vivo* expansion. Then, unbound hiPSC-EVs were washed out. Next, cells were resuspended in PBS and transplanted by retro-orbital plexus injection into γ-irradiated NOD/SCID mice (2×10^5^ of cells per mouse in the volume of 100 µl). Twenty-four hours before the cell transplantation, mice were irradiated with a double dose of 100 cGy at 4 h intervals (total dose 200 cGy). Irradiated animals with vehicle (PBS) administration served as a negative test control.

*Tissue harvesting and flow cytometry analysis*

After the transplantation mice were sacrificed at 48 h (analysis of homing) or 8 weeks (analysis of engraftment), the BM, peripheral blood (PB) and spleen were harvested. Each type of tissue was lysed with 1x BD Pharm Lyse buffer (BD Bioscience) to remove RBCs, washed in PBS, and resuspended in DMEM/F12 medium + 2% of FBS. The remaining TNCs were stained for 30 min at 4 °C with BV421-conjugated anti-human CD45 antibody (clone HI30; BD Bioscience). Prior to staining, anti-mouse CD16/CD32 antibody (BD Bioscience) was added for 5 min to block non-specific binding of anti-human antibodies to Fc receptors present on murine cells. Immediately before the analysis 7-AAD (BD Bioscience) was added to the cell samples. The presence of live (7-AAD-) human hematopoietic (hCD45^+^) cells in murine tissues was evaluated by LSRFortessa flow cytometer.

*CFC assay*

TNCs isolated from the BM of transplanted mice were counted, and then 1×10^6^ of cells were seeded onto the methylcellulose for CFC assay, analogously to the procedure described for CB-HSPCs. After 14 d, human hematopoietic colonies grown from transplanted cells that homed and engrafted to murine BM were counted.

**Statistical analysis**

Unless otherwise stated, all experiments were repeated at least three times. The exact number of repetitions (N) is indicated in the figure legends. Data on scatter plots present values for individual experimental repetitions with a black line representing the mean value. Data on the bar graphs present a mean ± standard deviation (SD). Graphs were performed using GraphPad Prism 5 Software (GraphPad Software Inc., San Diego, CA, USA). Statistical significance was calculated by Statistica Software (StatSoft, Tulsa, OK, USA) using a two-tailed Student’s *t*-test or a *t*-test with a fixed reference value of 1 (for the data expressed as –fold change) or 100% (for the data expressed as percentage of control). The value of p < 0.05 was considered significant and expressed on figures as black asterisks.

**Supplementary Figures**

**Supplementary Fig. 1 Flow cytometry phenotyping of RNASelect-negative fraction of hiPSC-EV samples.** **A** Representative dot plots of hiPSC-EVs samples stained with RNASelect dye and fluorescent antibodies, acquired by Apogee A50-Micro flow cytometer. In gating strategy, objects negative for RNASelect were taken into the analysis of surface antigens expression. The percentage of objects positive for the analyzed markers is shown in red gates. MALS-medium angle light scatter parameter, corresponding to the relative size of analyzed particles. **B** Quantitative data of RNASelect-negative hiPSC-EVs phenotyping, presented as the mean ± SD from experimental repetitions (N=3). **C** Percentage of expression of surface antigens on RNASelect-negative objects, expressed as a % of expression on RNASelect-positive fraction.

**Supplementary Fig. 2 Isolation of HSPCs-enriched CD34^+^ fraction from the CB.** CB units were lysed to remove RBCs. Next, CD34-enriched fraction was isolated by MACS, stained with appropriate fluorescent antibodies, and purified by FACS (**A**). CD45^dim^Lin^-^ cells (R2) were gated from FSC^low^/SSC^low^ lymphgate (R1), and then cells positive for CD34 were sorted from the gate R3. **B** Exemplary dot plot of CD34^+^ fraction purity after FACS. The numbers indicate the percentage of cells present in the gates as compared to the total cells on the dot plots.

**Supplementary Fig. 3 *Ex vivo* expansion of CB-HSPCs.** Purified cells were cultured for 14 d in the dedicated expansion medium containing hiPSC-EVs. **A** Morphology of expanding cells. Exemplary phase-contrast images of cells at subsequent time points of the culture as captured by an Olympus IX81 microscope. Scale bars indicate 50 µm. **B** Comparison of proliferation kinetics for cells expanded in the control medium or hiPSC-EVs medium (+hiPSC-EVs) expressed as a population doubling time. Data are presented as the mean ± SD (N=4).

**Supplementary Fig. 4 CFC assay.** CB-HSPCs were seeded in dedicated methylcellulose-based media for 14 days to form hematopoietic colonies. **A** Representative images of hematopoietic colonies: burst forming unit-erythroid (BFU-E); colony-forming unit-granulocyte-macrophage (CFU-GM); and colony-forming unit-granulocyte, erythrocyte, macrophage, megakaryocyte (CFU-GEMM) as captured by Olympus IX81 light microscope on 14 d of CFC assay. The scale bars indicate 200 µm. **B** Potential of CB-HSPCs expanded with hiPSC-EVs to form subsequent types of hematopoietic colonies. Cells were cultured for 14 d in the control medium (Ctrl) or hiPSC-EVs medium (+hiPSC-EVs). On selected day of expansion 5 × 10^2^ cells were seeded for CFC assay. Data on the graphs present the mean number of colonies (N=3). Table summarizes data as mean ± SD (N=3). **C** The effect of short-term treatment with hiPSC-EVs on the potential of expanded HSPCs to form particular colony types. Prior experiment, HSPCs were expanded for 7 d in the control medium and were subsequently treated with hiPSC-EVs for 2 h, 6 h, or 24 h. In selected timepoints 5 × 10^2^ cells were seeded for CFC assay. Data on the graphs present the mean number of colonies (N=4). Table summarizes data as mean ± SD (N=4). *P < 0.05 for control vs. hiPSC-EVs-treated cells, two-tailed Student’s *t*-test.

**Supplementary Tables**

**Supplementary Table 1. List of primers used in genetic analysis of CB-HSPCs followed by short-term treatment with hiPSC-EVs**. F: forward primer, R: a reverse primer.

| **Gene** | **Primer sequence** |
| --- | --- |
| *β2-microglobulin* | (F) AATGCGGCATCTTCAAAC (R) TGACTTTGTCACAGCCCAAGATA |
| *SCL* | (F) GGCTTTGTGTGAAGGCAGAGA (R) TCGCCAGCATGAACAGTGAT |
| *HOXB4* | (F) CCGATACCCAGCGAAAGC (R) TCAGTGAATGGGCACGAAAGA |
| *BCL-2* | (F) GAGTGACAGTGGATTGCAT  (R) CAGAATATCAGCCACCTCTT |

**Supplementary Table 2. List of genes analyzed by Hematopoiesis RT^2^ Profiler PCR Arrays**. The table contains short names of genes that were used in the manuscript.

| **Gene** | **Full gene name** |
| --- | --- |
| ***IL6ST*** | Interleukin 6 signal transducer (gp130) |
| ***CD3D*** | CD3 δ (CD3-TCR complex) |
| ***KIT*** | V-kit Hardy-Zuckerman 4 feline sarcoma viral oncogene homolog |
| ***JAG1*** | Jagged 1 |
| ***LRMP*** | Lymphoid-restricted membrane protein |
| ***VAV1*** | Vav 1 guanine nucleotide exchange factor |
| ***CD27*** | CD27 |
| ***RBPJ*** | Recombination signal binding protein for immunoglobulin kappa J region |
| ***ANGPT1*** | Angiopoietin 1 |
| ***CBFB*** | Core-binding factor β subunit |
| ***CEBPG*** | CCAAT/enhancer-binding protein (C/EBP), γ |
| ***INHBA*** | Inhibin α |
| ***TNFSF11*** | Tumor necrosis factor (ligand) superfamily, member 11 |
| ***ETV6*** | Ets variant 6 |
| ***NCOA6*** | Nuclear receptor coactivator 6 |
| ***CD164*** | CD164 |
| ***PECAM1*** | Platelet/endothelial cell adhesion molecule |
| ***IL31RA*** | Interleukin 31 receptor A |
| ***LEF1*** | Lymphoid enhancer-binding factor 1 |
| ***ASH2L*** | Ash2 (absent, small, or homeotic)-like (Drosophila) |
| ***GATA2*** | GATA binding protein 2 |
| ***VEGFA*** | Vascular endothelial growth factor A |
| ***STAT1*** | Signal transducer and activator of transcription 1 |
| ***GATA1*** | GATA binding protein 1 |
| ***CEBPE*** | CCAAT/enhancer-binding protein (C/EBP), ε |
| ***CD34*** | CD34 |
| ***FZD1*** | Frizzled family receptor 1 |
| ***HDAC7*** | Histone deacetylase 7 |
| ***HDAC9*** | Histone deacetylase 9 |
| ***CD44*** | CD44 |
| ***STAT3*** | Signal transducer and activator of transcription 3 |
| ***TAL1*** | T-cell acute lymphocytic leukemia 1 |
| ***PTPRC*** | Protein tyrosine phosphatase, receptor type C |
| ***CHST15*** | Carbohydrate (N-acetylgalactosamine 4-sulfate 6-O) sulfotransferase 15 |
| ***RUNX1*** | Runt-related transcription factor 1 |
| ***MAP4K1*** | Mitogen-activated protein kinase 1 |
| ***SFXN1*** | Sideroflexin 1 |
| ***HDAC5*** | Histone deacetylase 5 |
| ***CSF2*** | Colony stimulating factor 2 (granulocyte-macrophage) |
| ***LMO2*** | LIM domain only 2  (rhombotin-like 1) |
| ***CCR1*** | Chemokine (C-C motif) receptor 1 |
| ***ETS1*** | V-ets erythroblastosis virus E26 oncogene homolog 1 |
| ***CD14*** | CD14 |
| ***HDAC4*** | Histone deacetylase 4 |
| ***NOTCH1*** | NOTCH1 |
| ***NOTCH2*** | NOTCH2 |
| ***FUT10*** | Fucosyltransferase 10 |
| ***TLR4*** | Toll-like receptor 4 |
| ***IL12B*** | Interleukin 12B |
| ***PAX5*** | Paired box 5 |

**Supplementary Table 3. List of proteins analyzed by Proteome Profiler Arrays**. The table contains the short names of proteins that were used in the manuscript. Alternative protein names (based on the Uniprot database nomenclature) are included in the brackets.

| **Protein** | **Full protein name** |
| --- | --- |
| **HSP60** | Heat shock protein |
| **WNK1** | Serine/threonine-protein kinase Wnk1 |
| **Akt** | **Serine/threonine-protein kinase Akt** |
| **STAT** | Signal transducer and activator of transcription proteins |
| **RSK** | Ribosomal S6 kinase |
| **p53** | Cellular tumor antigen p53 |
| **p70 S6** | Ribosomal protein S6 kinase β-1 |
| **c-Jun** | **Transcription factor AP-1** |
| **PLCγ1** | Phospholipase Cγ1 |
| **Pyk2** | Protein-tyrosine kinase 2-β |
| **eNOS** | Endothelial nitric oxide synthase |
| **TOR (mTOR)** | Mammalian target of rapamycin |
| **Lyn** | **Tyrosine-protein kinase Lyn** |
| **PRAS40** | Proline-rich Akt substrate of 40 kDa |
| **Yes (YES1)** | **Tyrosine-protein kinase Yes** |
| **Src** | **Proto-oncogene tyrosine-protein kinase Src** |
| **Lck** | Lymphocyte cell-specific protein-tyrosine kinase |
| **Fyn** | **Tyrosine-protein kinase Fyn** |
| **CREB (CREB1)** | Cyclic AMP response element binding protein |
| **Chk-2 (CHEK2)** | Checkpoint kinase 2 |
| **Hck** | **Tyrosine-protein kinase Hck** |
| **Fgr** | **Tyrosine-protein kinase Fgr** |
| **PDGFRb** | Platelet-derived growth factor receptor β |
| **FAK (PTK2)** | Focal adhesion kinase |
| **CTNNB1** | β-catenin |
| **MSK (RPS6KA5)** | Mitogen and stress-activated protein kinase |
| **ERK (MAPK3)** | Extracellular signal regulated kinase |
| **EGFR** | **Epidermal growth factor receptor** |
| **p38α(MAPK14)** | **Mitogen-activated protein kinase 14** |
| **AMPK (PRKAA)** | 5'-AMP-activated protein kinase |
| **JNK (MAPK8)** | **Mitogen-activated protein kinase 8** |
| **GSK-3ab** | Glycogen synthase kinase 3ab |

**Supplementary Table 4. Influence of hiPSC-EVs on the hematopoietic differentiation of CB-HSPCs cells during *ex vivo* expansion.**  CD34^+^ cells purified from CB units were cultured in the dedicated control expansion medium (-hiPSC-EVs) or hiPSC-EVs medium (+hiPSC-EVs). The percentages of cells positive for particular surface antigens on indicated days of the expansion are presented as the mean ± SD (N=3).

| **Antigen** | **hiPSC-EVs** | **Day of expansion** | | | | |
| --- | --- | --- | --- | --- | --- | --- |
|  |  | **4** | **6** | **8** | **11** | **14** |
| **CD34** | **-** | 94.5 ± 4.7 | 79.6 ± 8.4 | 46.3 ± 13.2 | 17.0 ± 5.6 | 6.4 ± 1.6 |
|  | **+** | 94.4 ± 5.5 | 84.1 ± 11.7 | 46.3 ± 5.5 | 24.3 ± 7.6 | 5.5 ± 2.5 |
| **CD3** | **-** | 1.1 ± 0.5 | 2.5 ± 1.2 | 5.2 ± 2.2 | 10.5 ± 3.8 | 14.8 ± 2.0 |
|  | **+** | 1.5 ± 0.7 | 2.9 ± 0.5 | 6.9 ± 3.3 | 10.5 ± 2.0 | 19.3 ± 3.8 |
| **CD14** | **-** | 0.4 ± 0.4 | 2.2 ± 0.7 | 5.2 ± 1.5 | 7.3 ± 0.7 | 6.6 ± 2.1 |
|  | **+** | 0.5 ± 0.2 | 2.5 ± 0.4 | 4.1 ± 1.3 | 9.3 ± 1.1 | 7.6 ± 3.4 |
| **CD19** | **-** | 0.2 ± 0.1 | 0.8 ± 0.2 | 1.8 ± 0.4 | 3.3 ± 1.2 | 5.1 ± 1.1 |
|  | **+** | 0.5 ± 0.3 | 0.9 ± 0.2 | 2.3 ± 0.1 | 6.0 ± 2.5 | 7.2 ± 2.5 |
| **CD66b** | **-** | 0.2 ± 0.2 | 0.6 ± 0.2 | 0.9 ± 0.6 | 1.4 ± 0.5 | 2.1 ± 1.3 |
|  | **+** | 0.4 ± 0.1 | 1.2 ± 0.4 | 1.8 ± 0.2 | 2.4 ± 1.0 | 5.6 ± 4.0 |
| **CD235a** | **-** | 1.0 ± 0.6 | 5.4 ± 2.7 | 9.6 ± 4.1 | 8.9 ± 2.8 | 8.6 ± 3.7 |
|  | **+** | 1.8 ± 0.9 | 7.0 ± 1.8 | 7.4 ± 2.3 | 13.1 ± 3.0 | 11.7 ± 2.6 |

**Supplementary Table 5. Influence of hiPSC-EVs on the expression of hematopoietic-related genes in CB-HSPCs during *ex vivo* expansion.** CB-HSPCs purified from CB units were cultured in the dedicated control expansion medium or hiPSC-EVs medium. The relative changes in the level of particular transcripts were analyzed with the Hematopoiesis RT^2^ Profiler PCR Arrays. Data are expressed as a -fold change ± SD as compared to the control cells (expanded in medium without hiPSC-EVs). The table includes only those genes for which the expression changes were statistically significant (p < 0.05 compared to the control cells).

| **Day of expansion** | | | | | | | | |
| --- | --- | --- | --- | --- | --- | --- | --- | --- |
| **4** | | | | **8** | | | | |
| **Gene** | **–fold change** | | | **Gene** | **–fold change** | | | |
| ***JAG1*** | 0.43 | ± | 0.20 | ***GATA2*** | 0.22 | | ± | 0.07 |
| ***LRMP*** | 0.76 | ± | 0.46 | ***CEBPG*** | 0.22 | | ± | 0.00 |
| ***CD27*** | 0.79 | ± | 0.21 | ***HDAC5*** | 0.24 | | ± | 0.12 |
| ***ANGPT1*** | 0.92 | ± | 0.29 | ***PTPRC*** | 0.26 | | ± | 0.24 |
| ***NCOA6*** | 1.19 | ± | 0.48 | ***HDAC9*** | 0.31 | | ± | 0.28 |
| ***GATA2*** | 1.45 | ± | 0.44 | ***ANGPT1*** | 0.33 | | ± | 0.09 |
| ***VEGFA*** | 1.48 | ± | 0.29 | ***TLR4*** | 0.35 | | ± | 0.11 |
| ***GATA1*** | 1.49 | ± | 0.73 | ***IL12B*** | 0.35 | | ± | 0.20 |
| ***CEBPE*** | 1.49 | ± | 0.65 | ***LMO2*** | 0.37 | | ± | 0.20 |
| ***CD34*** | 1.51 | ± | 0.80 | ***JAG1*** | 0.40 | | ± | 0.15 |
| ***HDAC7*** | 1.55 | ± | 0.63 | ***STAT1*** | 0.41 | | ± | 0.48 |
| ***CD44*** | 1.66 | ± | 0.51 | ***SFXN1*** | 0.44 | | ± | 0.32 |
| ***PTPRC*** | 1.77 | ± | 0.31 | ***CSF2*** | 0.49 | | ± | 0.13 |
| ***CHST15*** | 1.94 | ± | 2.24 | ***CD44*** | 0.61 | | ± | 0.45 |
| ***RUNX1*** | 1.96 | ± | 0.08 | ***ETV6*** | 0.63 | | ± | 0.21 |
| ***MAP4K1*** | 2.09 | ± | 0.17 | ***FZD1*** | 0.67 | | ± | 0.60 |
| ***SFXN1*** | 2.14 | ± | 0.80 | ***KIT*** | 1.22 | | ± | 0.28 |
| ***HDAC5*** | 2.23 | ± | 0.35 | ***CBFB*** | 1.33 | | ± | 0.54 |
| ***CSF2*** | 2.45 | ± | 0.86 | ***NOTCH1*** | 1.37 | | ± | 0.91 |
| ***LMO2*** | 2.70 | ± | 0.33 | ***VEGFA*** | 1.54 | | ± | 1.30 |
| ***CCR1*** | 2.93 | ± | 1.42 | ***NOTCH2*** | 7.70 | | ± | 1.77 |
| ***ETS1*** | 3.09 | ± | 1.37 |  | |  | | |
| ***NOTCH1*** | 3.64 | ± | 1.15 |  | |  |  |  |
| ***NOTCH2*** | 3.90 | ± | 0.64 |  | |  |  |  |
| ***FUT10*** | 3.99 | ± | 0.80 |  | |  |  |  |
| ***TLR4*** | 11.20 | ± | 5.09 |  | |  |  |  |
| ***IL12B*** | 11.58 | ± | 4.69 |  | |  |  |  |
| ***PAX5*** | 11.66 | ± | 0.26 |  | |  |  |  |

**Supplementary Table 6. Influence of hiPSC-EVs on the level of kinases phosphorylation**. CB-HSPCs were incubated with hiPSC-EVs for 2 h. Phosphorylation of proteins was assessed by Proteome Profiler Arrays and expressed as a -fold change ± SD as compared to the control cells. The amino acid site of protein phosphorylation is included. Alternative protein names (based on the Uniprot database nomenclature) are included in the brackets.

| **Kinase** | **Phosphorylation site** | **-fold change** | | |
| --- | --- | --- | --- | --- |
| **HSP60** | -  - | 1.01  1.01 | ±  ± | 0.02  0.02 |
| **WNK1** | T60 | 1.08 | ± | 0.16 |
| **Akt** | T308 | 1.08 | ± | 0.05 |
| **STAT3** | S727 | 1.10 | ± | 0.32 |
| **RSK** | S380/S386/S377 | 1.12 | ± | 0.18 |
| **p53** | S46 | 1.14 | ± | 0.03 |
| **p70 S6** | T389 | 1.16 | ± | 0.06 |
| **p53** | S392 | 1.17 | ± | 0.09 |
| **p70 S6** | T421/S424 | 1.17 | ± | 0.08 |
| **STAT3** | Y705 | 1.21 | ± | 0.08 |
| **p53** | S15 | 1.35 | ± | 0.00 |
| **c-Jun** | S63 | 1.35 | ± | 0.09 |
| **PLCy1** | Y783 | 1.41 | ± | 0.05 |
| **Pyk2** | Y402 | 1.42 | ± | 0.02 |
| **eNOS** | S1177 | 1.47 | ± | 0.01 |
| **TOR (MTOR)** | S2448 | 1.71 | ± | 0.11 |
| **Lyn** | Y397 | 1.71 | ± | 0.14 |
| **PRAS40** | T246 | 1.72 | ± | 0.00 |
| **Yes (YES1)** | Y426 | 1.73 | ± | 0.04 |
| **Src** | Y419 | 1.77 | ± | 0.05 |
| **Lck** | Y394 | 1.82 | ± | 0.05 |
| **Fyn** | Y420 | 1.84 | ± | 0.04 |
| **CREB (CREB1)** | S133 | 1.85 | ± | 0.05 |
| **Chk-2 (CHEK2)** | T68 | 1.85 | ± | 0.04 |
| **Hck** | Y411 | 1.88 | ± | 0.04 |
| **HSP27 (HSPB1)** | S78/S82 | 1.91 | ± | 0.12 |
| **Fgr** | Y412 | 1.94 | ± | 0.08 |
| **PDGFRb** | Y751 | 2.06 | ± | 0.03 |
| **STAT2** | Y689 | 2.07 | ± | 0.01 |
| **FAK (PTK2)** | Y397 | 2.07 | ± | 0.04 |
| **b-catenin (CTNNB1)** | - | 2.11 | ± | 0.09 |
| **MSK (RPS6KA5)** | S376/S360 | 2.12 | ± | 0.12 |
| **ERK (MAPK3)** | T202/Y204, T185/Y187 | 2.12 | ± | 0.05 |
| **STAT5b** | Y699 | 2.26 | ± | 0.08 |
| **STAT5ab** | Y694/699 | 2.37 | ± | 0.04 |
| **Akt (AKT1)** | S473 | 2.37 | ± | 0.18 |
| **EGF R** | Y1086 | 2.42 | ± | 0.09 |
| **p38alpha (MAPK14)** | T180/Y182 | 2.42 | ± | 0.32 |
| **AMPK-a2 (PRKAA2)** | T172 | 2.52 | ± | 0.01 |
| **STAT6** | Y641 | 2.52 | ± | 0.06 |
| **AMPK a1 (PRKAA1)** | T183 | 2.64 | ± | 0.04 |
| **JNK (MAPK8)** | T183/Y185, T221/Y223 | 2.65 | ± | 0.06 |
| **STAT5a** | Y694 | 2.67 | ± | 0.02 |
| **GSK-3ab** | S21/S9 | 2.74 | ± | 0.09 |

**Supplementary Table 7. PANTHER Pathway analysis of protein phosphorylations upregulated in CB-HSPCs treated with hiPSC-EVs.** Proteins with a -fold change > 1.5 were included in the analysis (see also S5 Table for detailed list). A PANTHER Overrepresentation Test was performed. The list of PANTHER Pathways and the number of assigned proteins is presented. Only results with a False Discovery Rate (FDR) < 0.05 are displayed.

| [**PANTHER Pathways**](http://pantherdb.org/tools/compareToRefList.jsp?sortOrder=1&sortList=categories) | [**No. of proteins**](http://pantherdb.org/tools/compareToRefList.jsp?sortOrder=2&sortList=upload_1&sortField=num) **involved** | [**raw P value**](http://pantherdb.org/tools/compareToRefList.jsp?sortOrder=1&sortList=upload_1&sortField=pval) | [**FDR**](http://pantherdb.org/tools/compareToRefList.jsp?sortOrder=2&sortList=upload_1&sortField=fdr) |
| --- | --- | --- | --- |
| CCKR signaling map | 12 | 4.21·10^-18^ | 3.43·10^-16^ |
| Parkinson disease | 10 | 1.59·10^-16^ | 8.62·10^-15^ |
| PDGF signaling pathway | 10 | 5.60·10^-15^ | 2.28·10^-13^ |
| Gonadotropin-releasing hormone receptor pathway | 10 | 4.45·10^-13^ | 1.21·10^-11^ |
| EGF receptor signaling pathway | 9 | 2.13·10^-13^ | 6.96·10^-12^ |
| Angiogenesis | 9 | 1.32·10^-12^ | 3.06·10^-11^ |
| Interleukin signaling pathway | 7 | 3.57·10^-11^ | 7.28·10^-10^ |
| Cadherin signaling pathway | 6 | 6.63·10^-08^ | 8.32·10^-07^ |
| JAK/STAT signaling pathway | 5 | 7.37·10^-11^ | 1.33·10^-09^ |
| VEGF signaling pathway | 5 | 4.01·10^-08^ | 6.54·10^-07^ |
| Ras Pathway | 5 | 5.99·10^-08^ | 8.13·10^-07^ |
| Integrin signalling pathway | 5 | 5.33·10^-06^ | 5.43·10^-05^ |
| p53 pathway by glucose deprivation | 4 | 4.29·10^-08^ | 6.35·10^-07^ |
| p38 MAPK pathway | 4 | 3.92·10^-07^ | 4.56·10^-06^ |
| B cell activation | 4 | 2.55·10^-06^ | 2.77·10^-05^ |
| T cell activation | 4 | 7.31·10^-06^ | 7.01·10^-05^ |
| Apoptosis signaling pathway | 4 | 2.10·10^-05^ | 1.80·10^-04^ |
| FGF signaling pathway | 4 | 2.30·10^-05^ | 1.88·10^-04^ |
| Interferon-gamma signaling pathway | 3 | 1.02·10^-05^ | 9.20·10^-05^ |
| p53 pathway feedback loops 2 | 3 | 4.98·10^-05^ | 3.87·10^-04^ |
| Toll receptor signaling pathway | 3 | 7.54·10^-05^ | 5.59·10^-04^ |
| Alzheimer disease-amyloid secretase pathway | 3 | 1.18·10^-04^ | 8.35·10^-04^ |
| TGF-beta signaling pathway | 3 | 3.12·10^-04^ | 2.12·10^-03^ |
| Inflammation mediated by chemokine and cytokine signaling pathway | 3 | 4.74·10^-03^ | 2.58·10^-02^ |
| Insulin/IGF pathway-mitogen activated protein kinase kinase/MAP kinase cascade | 2 | 9.86·10^-04^ | 6.43·10^-03^ |
| Hypoxia response via HIF activation | 2 | 9.86·10^-04^ | 6.18·10^-03^ |
| FAS signaling pathway | 2 | 1.04·10^-03^ | 6.30·10^-03^ |
| Insulin/IGF pathway-protein kinase B signaling cascade | 2 | 1.49·10^-03^ | 8.66·10^-03^ |
| Oxidative stress response | 2 | 2.87·10^-03^ | 1.62·10^-02^ |
| Endothelin signaling pathway | 2 | 5.94·10^-03^ | 3.12·10^-02^ |
| p53 pathway | 2 | 6.48·10^-03^ | 3.30·10^-02^ |

**Supplementary Movies legends**

**Supplementary Movie 1.** **hiPSC-EVs enter into CB-HSPCs *in vitro***. Visualization of copGFP+ hiPSC-EVs internalization into CB-HSPCs after 2 h of incubation. Cell images were captured by Leica DMI6000B microscope with 100x NA-1.47 oil immersion objective. A composition of a few representative view positions, showing Z-stacks images of CB-HSPCs, as merged images for differential interference contrast module and fluorescence channel for copGFP. The cells were scanned along Z-axis (slices) starting from the bottom to the top of the cells, with 0.175 µm steps (as maximal resolution of used 100x/1.47 oil objective). Note the copGFP-positive EVs gradually appearing inside the cells during the changes of focal deepness.
